# Progress of Multi Functional Properties of Organic-Inorganic Hybrid System, A[Fe^II^Fe^III^X_3_] (A = (n-C_n_H_2n+1_)_4_N, Spiropyran; X = C_2_O_2_S_2_, C_2_OS_3_, C_2_O_3_S)

**DOI:** 10.3390/ma3053141

**Published:** 2010-05-11

**Authors:** Norimichi Kojima, Masaya Enomoto, Noriyuki Kida, Koichi Kagesawa

**Affiliations:** Graduate school of Arts and Sciences, The University of Tokyo, Komaba 3-8-1, Meguro-ku, Tokyo 153-8902, Japan; E-Mails: menomoto@rs.kagu.tus.ac.jp (M.E.); 2118830@cc.m-kagaku.co.jp (N.K.); cc087906@mail.ecc.u-tokyo.ac.jp (K.K.)

**Keywords:** concerted phenomenon, charge transfer phase transition, mixed valence, ferromagnetism, photo-isomerization, photo-induced phase transition, valence fluctuation

## Abstract

In the case of mixed-valence systems whose spin states are situated in the spin crossover region, new types of conjugated phenomena coupled with spin and charge are expected. From this viewpoint, we have investigated the multifunctional properties coupled with spin, charge and photon for the organic-inorganic hybrid system, A[Fe^II^Fe^III^X_3_](A = (*n*-C_n_H_2n+1_)_4_N, spiropyran; X = dto(C_2_O_2_S_2_), tto(C_2_OS_3_), mto(C_2_O_3_S)). A[Fe^II^Fe^III^(dto)_3_] and A[Fe^II^Fe^III^(tto)_3_] undergo the ferromagnetic phase transitions, while A[Fe^II^Fe^III^(mto)_3_] undergoes a ferrimagnetic transition. In (*n*-C_n_H_2n+1_)_4_N [Fe^II^Fe^III^(dto)_3_](n = 3,4), a new type of phase transition called charge transfer phase transition (CTPT) takes place around 120 K, where the thermally induced charge transfer between Fe^II^ and Fe^III^ occurs reversibly. At the CTPT, the iron valence state dynamically fluctuated with a frequency of about 0.1 MHz, which was confirmed by means of muon spin relaxation. The charge transfer phase transition and the ferromagnetic transition for (*n*-C_n_H_2n+1_)_4_N[Fe^II^Fe^III^(dto)_3_] remarkably depend on the size of intercalated cation. In the case of (SP)[Fe^II^Fe^III^(dto)_3_](SP = spiropyran), the photoinduced isomerization of SP under UV irradiation induces the charge transfer phase transition in the [Fe^II^Fe^III^(dto)_3_] layer and the remarkable change of the ferromagnetic transition temperature. In the case of (*n*-C_n_H_2n+1_)_4_N[Fe^II^Fe^III^(mto)_3_](mto = C_2_O_3_S), a rapid spin equilibrium between the high-spin state (*S* = 5/2) and the low-spin state (*S* = 1/2) at the Fe^III^O_3_S_3_ site takes place in a wide temperature range, which induces the valence fluctuation of the FeS_3_O_3_ and FeO_6_ sites through the ferromagnetic coupling between the low spin state (*S* = 1/2) of the Fe^III^S_3_O_3_ site and the high spin state (*S* = 2) of the Fe^II^O_6_ site.

## 1. Introduction

One of the most important targets in current research in the field of molecular solids is investigating the multifunctional properties coupled with transport, optical or magnetic properties. Assembled hetero-molecular systems such as organic-inorganic hybrid system have the possibility to undergo significant concert phenomena as a whole system through the hetero-molecular interaction between the constituent elements. In the case of conjugated phenomena coupled with transport and magnetic properties, for example, a metallic organic conductor coexisting with ferromagnetism for (BEDT-TTF)_3_ [Mn(II)Cr(III)(C_2_O_4_)_3_] (BEDT-TTF = 4,5-bis(ethylenedithio)tetrathiafulvalene) [1], a field-induced superconductivity for λ-(BETS)_2_FeCl_4_ (BETS = 4,5-bis(ethylenedithio) tetraselenafulvalene) [2,3], or the competition of superconducting phase and insulating antiferromagnetic phase for λ-(BETS)_2_Ga_1-x_Fe_x_Cl_4_ [4] have been reported. In the case of phenomena coupled with optical and magnetic properties, the light induced excited spin state trapping (LIESST) in spin-crossover complexes [5,6,7], or the photo-induced magnetism in transition metal cyanides [8,9,10,11,12,13] have been reported. In the case of phenomena coupled with optical and transport properties, the photo-induced valence transition for halogen-bridged gold mixed-valence complexes, Cs_2_[Au(I)Au(III)Br_6_] [14,15], iodine-bridged binuclear Pt complexes [16], or the photo-induced transition between the metallic and insulating phases for organic salts [17,18,19] have been reported.

Among various multifunctional materials, organic-inorganic hybrid systems are the leading candidates as field-sensitive multifunctional materials. In particular, organic-inorganic hybrid systems such as layered double hydroxides (LDHs) [20,21,22,23,24], oxalato-bridged bimetal complexes A[M^II^M’^III^(ox)_3_] (A = cation, M, M’ = metal, ox = C_2_O_4_) [25,26,27,28,29,30], dithiooxalato-bridged bimetal complexes A[M^II^M’^III^(dto)_3_] (A = cation, M, M’ = metal, dto = C_2_S_2_O_2_) [31,32,33,34,35,36,37,38,39,40,41,42], perovskite-type metal halides A_2_M^II^X_4_ (A = cation, M = metal, X = halogen) [43,44,45], magnetic vesicles [46,47,48,49], or magnetic thin films [50,51,52] provide an excellent opportunity to control their magnetic properties by the intercalation of various molecules.

**Figure 1:**
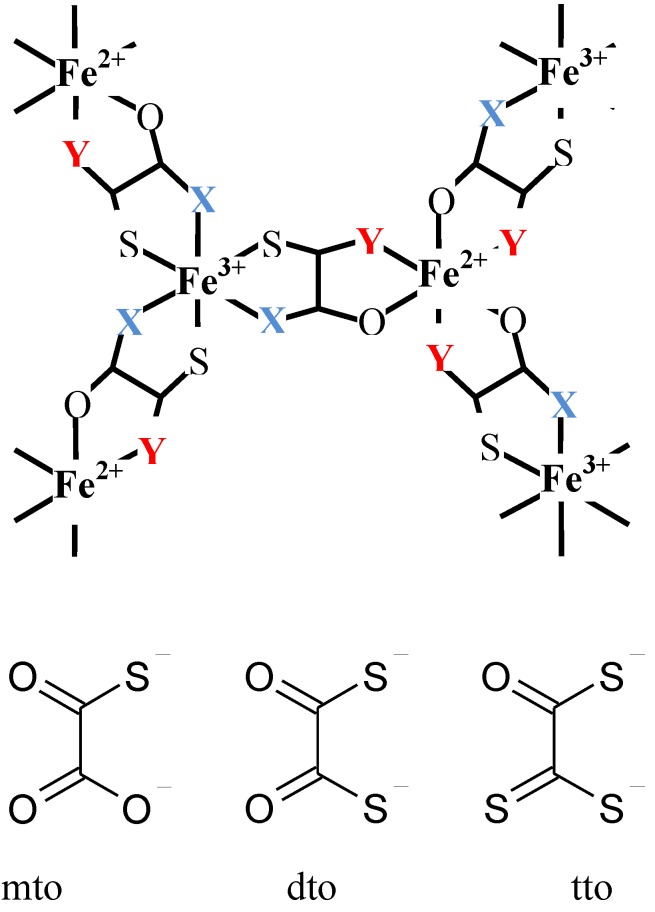
Schematic feature of the iron mixed valence complexes, [Fe^II^Fe^III^X_3_](X = mto, dto, tto), with the bridging ligand of oxalato derivatives.

In connection with magnetic materials, in the case of mixed-valence systems whose spin states are situated in the spin crossover region, it is expected that new types of conjugated phenomena coupled with spin and charge take place between different metal ions in order to minimize the free energy in the whole system. Based on this viewpoint, we have developed organic-inorganic hybrid systems, A[Fe^II^Fe^III^X_3_](A = (*n*-C_n_H_2n+1_)_4_N, spiropyran; X = dto(C_2_O_2_S_2_), tto(C_2_OS_3_), mto(C_2_O_3_S)), and have investigated their multifunctional properties coupled with spin, charge and photon. Figure 1 shows the schematic feature of the iron mixed-valence complex with the bridging ligand of oxalato derivatives, [Fe^II^Fe^III^X_3_](X = dto, tto and mto).

In the case of (*n*-C_n_H_2n+1_)_4_N[Fe^II^Fe^III^(dto)_3_], we have discovered a new type of phase transition called charge transfer phase transition (CTPT) where the thermally induced charge transfer between Fe^II^ and Fe^III^ occurs reversibly [33,34]. At the CTPT, we have found the iron valence fluctuation with a frequency of about 0.1 MHz by means of muon spin relaxation [41]. (*n*-C_n_H_2n+1_)_4_N[Fe^II^Fe^III^(dto)_3_] (n = 3–6) undergo the ferromagnetic phase transitions [40]. The charge transfer phase transition and the ferromagnetic transition in (*n*-C_n_H_2n+1_)_4_N[Fe^II^Fe^III^(dto)_3_] remarkably depend on the size of intercalated cation [40]. Based on this result, we have succeeded in controlling the charge transfer phase transition and the ferromagnetism for (SP)[Fe^II^Fe^III^(dto)_3_] (SP = spiropyran) by means of photoisomerization of SP [42].

In the case of (*n*-C_3_H_7_)_4_N[Fe^II^Fe^III^(tto)_3_](tto = C_2_OS_3_), the LTP with Fe^II^(*S* = 0) and Fe^III^(*S* = 5/2) is stable in the whole measuring temperature range between 300 K and 5 K. This complex undergoes the ferromagnetic transition at 9.5 K.

In the case of (*n*-C_n_H_2n+1_)_4_N[Fe^II^Fe^III^(mto)_3_] (mto = C_2_O_3_S), we have found that a rapid spin equilibrium between the high-spin state (*S* = 5/2) and the low-spin state (*S* = 1/2) at the Fe^III^O_3_S_3_ site takes place in a wide temperature range, which induces the valence fluctuation of the FeS_3_O_3_ and FeO_6_ sites through the ferromagnetic coupling between the low spin state (*S* = 1/2) of the Fe^III^S_3_O_3_ site and the high spin state (*S* = 2) of the Fe^II^O_6_ site [53].

In this paper, we review various multifunctional properties coupled with spin, charge and photon for the organic-inorganic hybrid system, A[Fe^II^Fe^III^X_3_] (A = (*n*-C_n_H_2n+1_)_4_N, spiropyran; X = C_2_O_2_S_2_, C_2_OS_3_, C_2_O_3_S).

## 2. A[Fe^II^Fe^III^(dto)_3_] (A = (*n*-C_n_H_2n+1_)_4_N, spiropyran; dto = C_2_O_2_S_2_)

As mentioned in the chapter 1, the charge transfer phase transition (CTPT) is a new type of conjugated phenomenon coupled with spins and charges wherein a thermally induced metal-to-metal electron transfer occurs to minimize the free energy in the whole system [33]. As a result of electron transfer, the spin degeneracy changes without spin transition, which is quite different from a classical spin crossover phenomenon. This spin-entropy effect has an important role in the driving force of the CTPT. In the cases of the iron mixed-valence complexes, (*n*-C_3_H_7_)_4_N[Fe^II^Fe^III^(dto)_3_] and (*n*-C_4_H_9_)_4_N[Fe^II^Fe^III^(dto)_3_] show a novel CTPT between Fe^II^ and Fe^III^ in the [Fe^II^Fe^III^(dto)_3_] layer. The existence of the CTPT depends on the size of cationic intercalant [39,40].

### 2.1. Crystal structure

**Figure 2:**
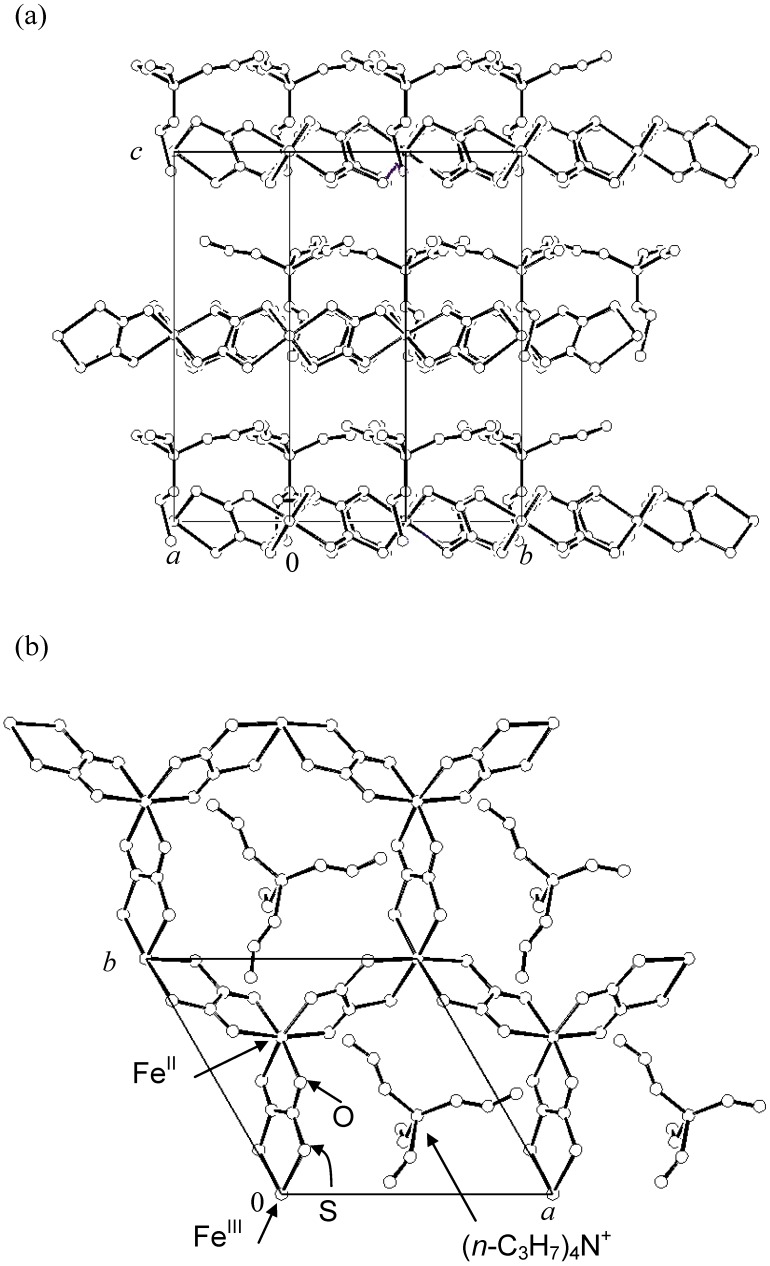
Crystal structure of (*n*-C_3_H_7_)_4_N[Fe^II^Fe^III^(dto)_3_] at room temperature. **(a)** [210] projection, **(b)** [001] projection [38].

Single crystals of (*n*-C_3_H_7_)_4_N[Fe^II^Fe^III^(dto)_3_] were grown by a diffusion method with the solvent of methanol–water mixture, where KBa[Fe^III^(dto)_3_]·3H_2_O, FeCl_2_·4H_2_O were placed at one bottom of H-tube, while (*n*-C_3_H_7_)_4_NBr was kept at the other bottom. The single-crystal X-ray structural analysis reveals the existence of a two-dimensional (2D) honeycomb network structure of [Fe^II^Fe^III^(dto)_3_] with intercalated counter cation as shown in Figure 2 [38]. The lattice parameters are as follows; space group *P*6_3_, *a* = 10.0618(5) Å, *c* = 16.0424(7) Å , *V* = 1406.54(12) Å^3^, *Z* = 2. The characteristic bond lengths of Fe-O and Fe-S for the as-prepared sample are in good agreement with those for Fe^II^-O and Fe^III^-S, respectively [37,54]. In this complex, the Fe^II^ and Fe^III^ atoms are alternately bridged by dithiooxalato molecules, which forms the 2D honeycomb network structure of [Fe^II^Fe^III^(dto)_3_]. The (*n*-C_3_H_7_)_4_N cation layer is intercalated between two adjacent [Fe^II^Fe^III^(dto)_3_] layers. This structure is common in hetero-metal oxalato and dithiooxalato complexes [37,55].

**Figure 3:**
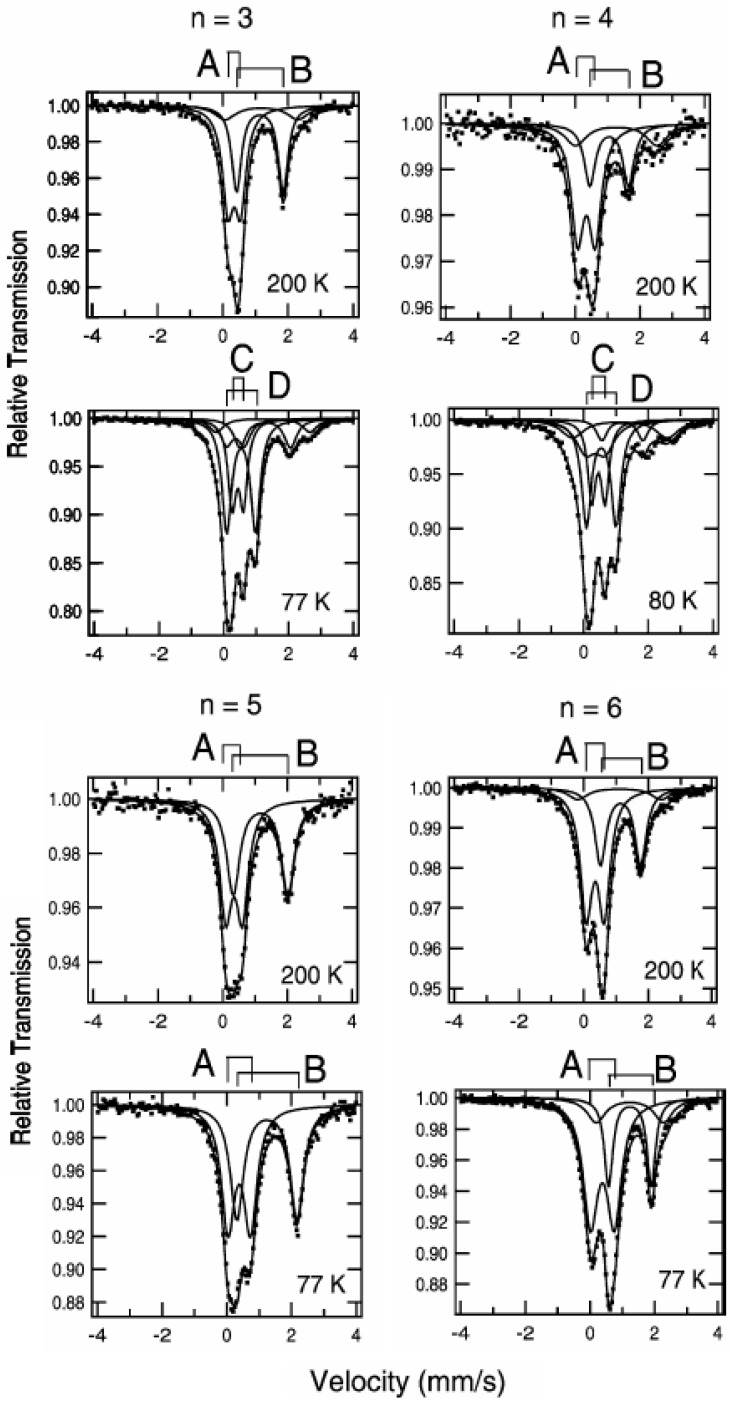
^57^Fe Mössbauer spectra for (*n*-C_n_H_2n+1_)_4_N[Fe^II^Fe^III^(dto)_3_](n = 3 – 6). A: Fe^III^(*S* = 1/2), B: Fe^II^(*S* = 2), C: Fe^II^(*S* = 0), D: Fe^III^(*S* = 5/2) [39].

As mentioned above, Fe^II^ and Fe^III^ sites in (*n*-C_3_H_7_)_4_N[Fe^II^Fe^III^(dto)_3_] are coordinated by six O and six S atoms at room temperature, respectively. Moreover, the ^57^Fe Mössbauer spectroscopy specifies their spin states. Figure 3 shows the ^57^Fe Mössbauer spectra for (*n*-C_n_H_2n+1_)_4_N[Fe^II^Fe^III^(dto)_3_](n = 3-6) [39]. The assignment of the spectra A, B, C and D were confirmed by the ^57^Fe Mössbauer spectra of (*n*-C_3_H_7_)_4_N[^57^Fe^II^Fe^III^(dto)_3_] and (*n*-C_3_H_7_)_4_N[Fe^II 57^Fe^III^(dto)_3_]. At 200 K, the line profiles of all the complexes are quite similar to each other. The isomer shift (*IS*) and the quadrupole splitting (*QS*) of the spectrum A at 200 K for (*n*-C_n_H_2n+1_)_4_N[Fe^II^Fe^III^(dto)_3_](n = 3-6) are quite similar to those (*IS* = 0.33 mm/s, *QS* = 0.35 mm/s at 196 K) of the ^57^Fe Mössbauer spectrum for the Fe^III^(*S* = 1/2) site in KBa[Fe^III^(dto)_3_]·3H_2_O [56], where the Fe^III^ site is coordinated by six S atoms. On the other hand, the *IS* and *QS* of the spectrum B at 200 K are quite similar to those (*IS* = 1.235 mm/s, *QS* = 1.42 mm/s at 190 K) of the ^57^Fe Mössbauer spectrum for the Fe^II^(*S* = 2) site in (*n*-C_4_H_9_)_4_N[Fe^II^Fe^III^(ox)_3_](ox = oxalato) [57], where the Fe^II^ site is coordinated by six O atoms. Therefore, it is concluded that the Fe^II^(*S* = 2) and Fe^III^(*S* = 1/2) sites in (*n*-C_n_H_2n+1_)_4_N[Fe^II^Fe^III^(dto)_3_](n = 3-6) are coordinated by six O atoms and six S atoms, respectively. In the cases of n = 3, 4 and 6, besides the ^57^Fe Mössbauer spectra due to the Fe^III^S_6_ and Fe^II^O_6_ octahedra, a weak spectrum with two branches at about 0.0 mm/s and 2.5 mm/s is observed, which is attributed to the high-spin state(*S* = 2) of Fe^II^O_4_S_2_ octahedron caused by the linkage isomerization on precipitation process.

### 2.2. Charge transfer phase transition (CTPT) and ferromagnetism

In the cases of n = 3 and 4, at 77 K (80 K for n = 4), the spectra A and B decrease by about 80%. Instead of these spectra, the spectra C and D appear. The *IS* and *QS* of the spectrum C for n = 3 and 4 are similar to those (*IS* = 0.325 mm/s, *QS* = 0.39 mm/s) of the Fe^II^(*S* = 0) site in [Fe^II^(bipy)_3_](ClO_4_)_2_ (bipy = 2,2’-bipyridine) [58]. On the other hand, the *IS* and QS of the spectrum D for n = 3 and 4 are similar to those (*IS* = 0.486, *QS* = 0.64 at 90 K) of the ^57^Fe Mössbauer spectrum for the Fe^III^(*S* = 5/2) site in (*n*-C_4_H_9_)_4_N [Fe^II^Fe^III^(ox)_3_] [57].

**Figure 4:**
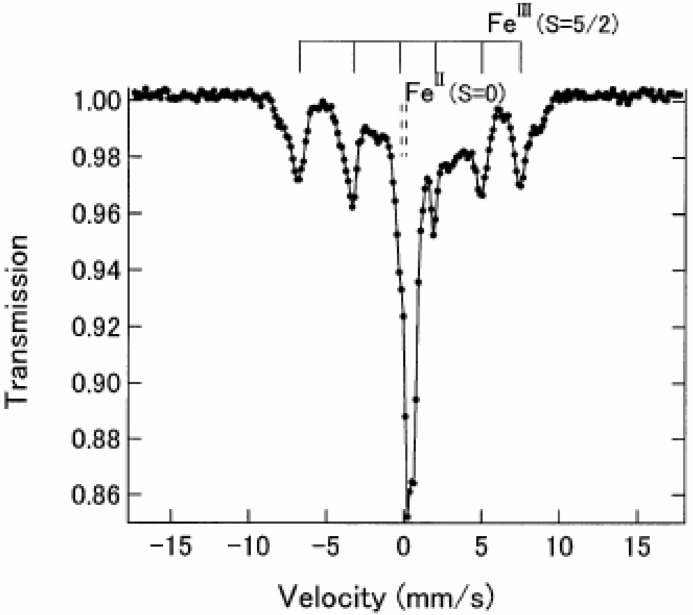
^57^Fe Mössbauer spectrum of (*n*-C_3_H_7_)_4_N[Fe^II^Fe^III^(dto)_3_] at 4.2 K. Solid and broken lines show the spectral peak positions of the ^57^Fe Mössbauer spectrum corresponding to the Fe^III^(*S* = 5/2) and Fe^II^ (*S* = 0) sites, respectively [32].

As shown later in this section, n = 3 undergoes the ferromagnetic transition at 7 K [32]. Figure 4 shows the ^57^Fe Mössbauer spectra in the ferromagnetically ordered phase of n = 3 [32]. They are well resolved and show a superposition of a central peak and a magnetically split spectrum with six branches. The line profile of ^57^Fe Mössbauer spectrum for n = 3 in the ferromagnetically ordered phase is quite similar to that of the ^57^Fe Mössbauer spectrum for Prussian blue, (Fe^III^[Fe^II^(CN)_6_]_3_) below *T*_C_ ( = 5.5 K) [59]. In the case of Prussian blue, the spin state of the Fe^II^ site coordinated by six C atoms is the low spin state (*S* = 0), and that of the Fe^III^ site coordinated by N atoms is the high spin state (*S* = 5/2). The estimated internal magnetic fields for Fe^II^ (*S* = 0) and Fe^III^ (*S* = 5/2) in Fe^III^[Fe^II^(CN)_6_]_3_ at 1.6 K are 0 and 540 kOe, respectively [59]. Comparing the internal magnetic fields for the Fe^II^ and Fe^III^ sites in n = 3, the ^57^Fe Mössbauer spectrum with six split branches induced by the internal magnetic field of 446 kOe corresponds to that for the Fe^III^ site with high spin state (*S* = 5/2), and the central peak without internal magnetic field corresponds to that for the Fe^II^ site with low spin state (*S* = 0). Taking into account the obtained *IS* and *QS* of the ^57^Fe Mössbauer spectra for the Fe^II^ and Fe^III^ sites at 4.2 K, the ^57^Fe Mössbauer spectra of n = 3 at 77 K can be reproduced as shown in Figure 3.

From the ^57^Fe Mössbauer spectra for (*n*-C_n_H_2n+1_)_4_N[Fe^II^Fe^III^(dto)_3_](n = 3 and 4), it is obvious that the charge transfer phase transition (CTPT) takes place between 200 K and 77 K for n = 3 and 4. The coexistence of the higher and lower temperature phases at 77 K is typical of first order phase transition, which reflects on the thermal hysteresis in magnetic susceptibility [40]. From the analysis of heat capacity, the critical temperature of the CTPT was determined at 122.4 K for n = 3 [33]. In the high temperature phase, the spin configuration becomes Fe^II^(S = 2)-Fe^III^(S = 1/2) for as-prepared sample. Below 120 K, on the other hand, the spin state of the Fe^II^ coordinated by six S atoms should be *S* = 0 and that of the Fe^III^ coordinated by six O atoms should be *S* = 5/2, which is able to explain the values of *IS*, *QS* and *H*_int_ of the ^57^Fe Mössbauer spectra in the low temperature phase.

**Figure 5:**
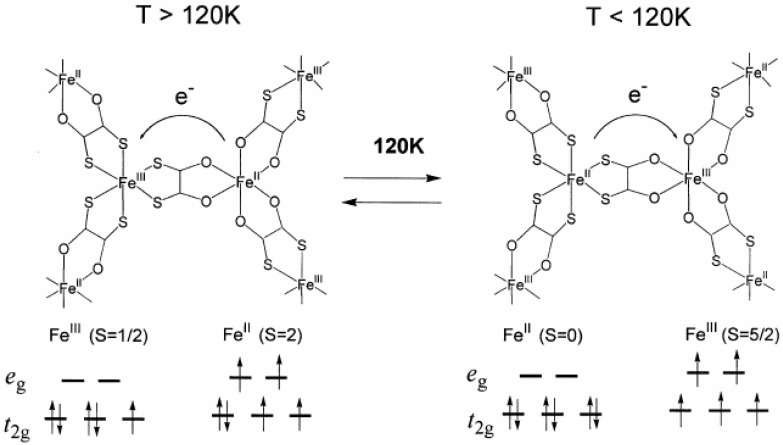
Schematic feature of the charge transfer phase transition in (*n*-C_3_H_7_)_4_N[Fe^II^Fe^III^(dto)_3_] [32].

Therefore, it is concluded that (*n*-C_3_H_7_)_4_N[Fe^II^Fe^III^(dto)_3_] undergoes a thermally induced CTPT at about 120 K where the electron transfer occurs reversibly between the *t*_2g_ orbitals of the Fe^II^ and Fe^III^ sites, which is schematically shown in Figure 5. The driving force responsible for the CTPT is the difference in spin entropy between the high temperature phase (HTP) and the low temperature one (LTP). It should be noted that the spin entropy in the high temperature phase is R ln(2 × 5) = 19.15 J·K^-1^·mol^-1^and that in the low temperature phase is R ln(1 × 6) = 14.90 J·K^-1^·mol^-1^, where R is the gas constant. Therefore, the spin-entropy gain expected from the electron transfer from the *t*_2g_ orbital of the Fe^II^ site to that of the Fe^III^ site is estimated at 4.25 J·K^-1^·mol^-1^. Since the observed entropy gain at the CTPT in (*n*-C_3_H_7_)_4_N[Fe^II^Fe^III^(dto)_3_] is 9.20 J·K^-1^·mol^-1^ [33], the entropy change originating from the lattice vibration is quite smaller than that for classical spin-crossover transition. For example, about 35 J·K^-1^·mol^-1^ was estimated for the vibrational contribution to the entropy change in the spin crossover transition in [Fe(phen)_2_(NCS)_2_] [60].

In the cases of n = 5 and 6, the line profile of the ^57^Fe Mössbauer spectra remains unchanged between 200 K and 77 K, which implies that the charge transfer phase transition does not take place for n = 5 and 6. In fact, the higher temperature phase exists between 300 K and 4 K for n = 5 and 6. The charge transfer phase transition is sensitive to the size of 2D honeycomb network structure. The increase of cation size expands the honeycomb ring, which presumably stabilizes the higher temperature phase.

**Figure 6:**
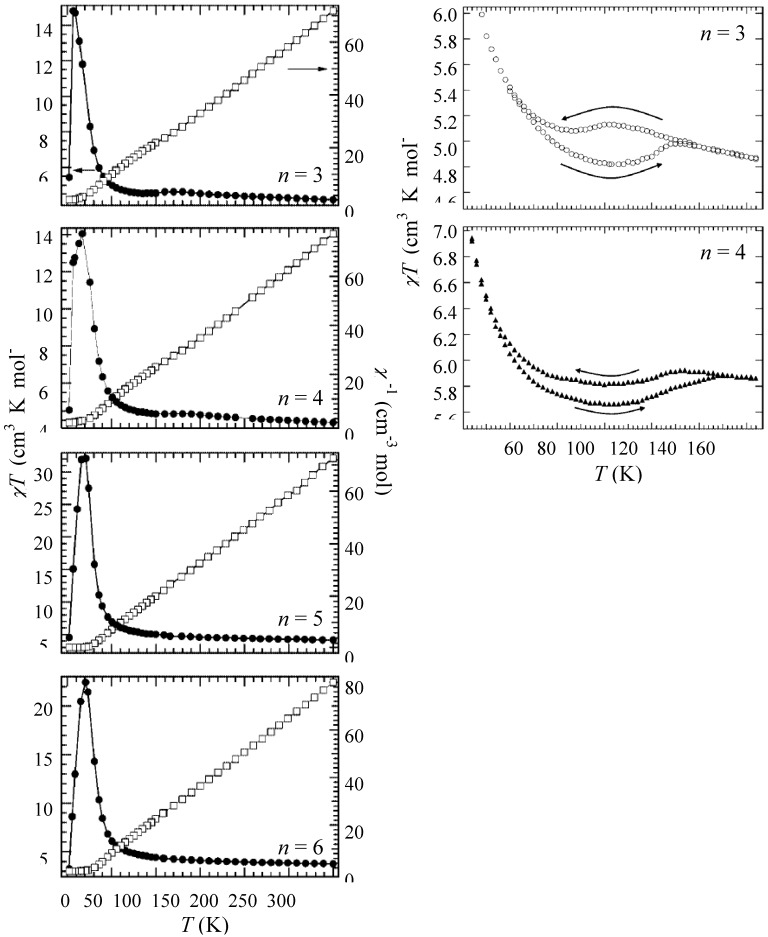
Temperature dependence of the magnetic susceptibility multiplied by temperature (χ*T*: ●) and the inverse susceptibility (χ^–1^: ○) of (*n*-C_n_H_2n+1_)_4_N[Fe^II^Fe^III^(dto)_3_](n = 3 – 6) [40].

Figure 6 shows the χ*T* and the inverse magnetic susceptibility (χ^–1^) as a function of temperature for (*n*-C_n_H_2n+1_)_4_N[Fe^II^Fe^III^(dto)_3_](n = 3-6) [40]. The magnetic susceptibilities for all the complexes obey the Curie-Weiss law, χ^–1^ = (*T*-θ) / *T*, in the range of 150-300 K. The Weiss constants of n = 3 – 6 are estimated at +12 K, +18 K, +23 K and +21 K, respectively. All the positive Weiss constants imply the ferromagnetic interaction between Fe^II^ and Fe^III^ in (*n*-C_n_H_2n+1_)_4_N[Fe^II^Fe^III^(dto)_3_]. In the cases of n = 3 and 4, reflecting the CTPT, a small bump appears in the χ*T* curve and the slope of χ^–1^ changes around 120 K and 140 K for n = 3 and n = 4, respectively. In this temperature region, a thermal hysteresis loop appears between 60-130 K and 50-150 K for n = 3 and n = 4, respectively.

**Figure 7:**
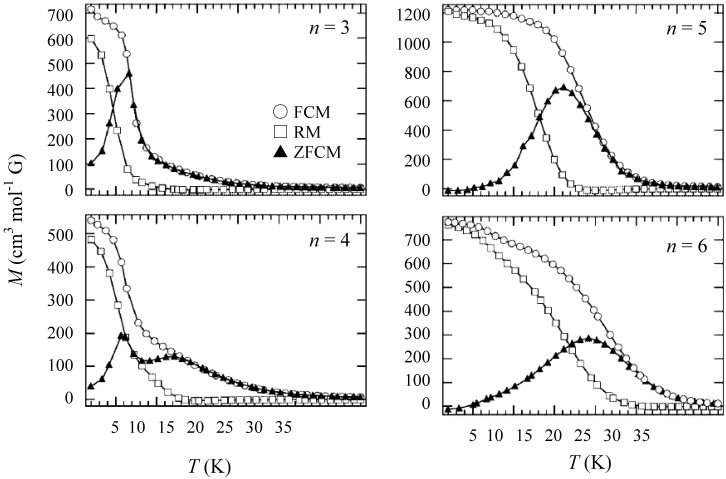
Temperature dependence of the field cooled magnetization (FCM), the remnant magnetization (RM), and the zero-field cooled magnetization (ZFCM) for (*n*-C_n_H_2n+1_)_4_N[Fe^II^Fe^III^(dto)_3_](n = 3–6). Applied magnetic field, *H* = 30 Oe [40].

**Figure 8:**
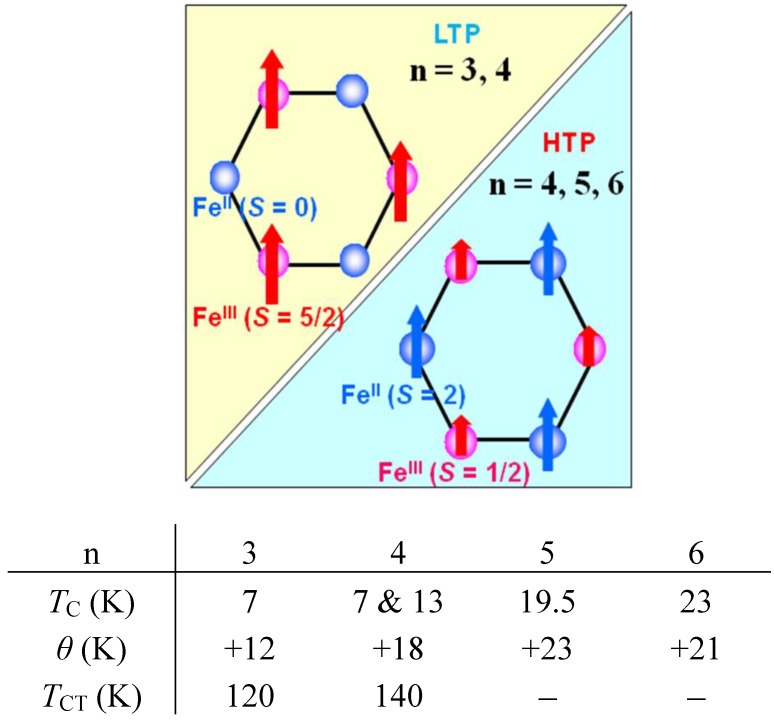
Schematical feature of ferromagnetism and the magnetic parameters for (*n*-C_n_H_2n+1_)_4_N[Fe^II^Fe^III^(dto)_3_](n = 3–6). *T*_CT_ denotes the critical temperature of the charge transfer phase transition.

In order to confirm the ferromagnetic phase transition, we have investigated the field-cooled magnetization (FCM), the remnant magnetization (RM) and the zero-field cooled magnetization (ZFCM) for n = 3–6, which are shown in Figure 7 [40]. The FCM curve was obtained on cooling with an external magnetic field of 30 Oe. After the measurement of FCM, the magnetic field was switched off at 2 K and then the RM was measured from 2 K to 35 K. After cooling from 300 K to 2 K with complete zero external field, the external field of 30 Oe was switched on at 2 K, then the ZFCM was measured from 2 K to 35 K. The RM corresponds to the spontaneous magnetization. Below the ferromagnetic transition, the ZFCM is smaller than the FCM, which is due to the fact that the applied magnetic field of 30 Oe is too weak to move the magnetic domain walls below the Curie temperature. The ZFCM and FCM curves meet each other at the Curie temperature where the hysteresis disappears. In this way, the Curie temperature is evaluated. More precisely, the Curie temperature were estimated at 7, (7 and 13), 19.5 and 23 K for n =3-6, respectively, from the heat capacity measurements for n = 3 and 4, [33] and from the analysis of Arrott plot for n = 5 and 6 [40]. In the case of n = 4, the ZFCM has two peaks at 7 K and 13 K, and the RM vanishes at 13 K, which implies that the LTP and HTP coexist even at 2 K and these phases undergo the ferromagnetic phase transitions at *T*_C_ = 7 K and *T*_C_ = 13 K, respectively. The critical temperature of CTPT, the Weiss constant and the Curie temperature for (*n*-C_n_H_2n+1_)_4_N[Fe^II^Fe^III^(dto)_3_](n = 3-6) are summarized in Figure 8.

### 2.3. Cation size effect on the crystal structure

**Figure 9:**
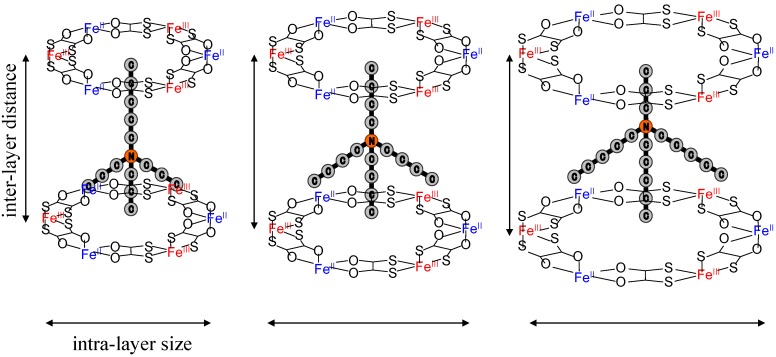
Schematic representation of a part of two-dimensional network structure modified by axially symmetrical counter cations in (*n*-C_m_H_2m+1_)_3_(*n*-C_n_H_2n+1_)N[Fe^II^Fe^III^(dto)_3_] (*m* + *n* = 8) [61].

As mentioned in the previous section, the CTPT of (*n*-C_n_H_2n+1_)_4_N[Fe^II^Fe^III^(dto)_3_] exhibits a significant dependence on the size of counter cation. In order to investigate the more detailed structural factor that controls the CTPT, we have synthesized iron mixed valence complexes with various size of counter cations, (*n*-C_m_H_2m+1_)_3_(*n*-C_n_H_2n+1_)N[Fe^II^Fe^III^(dto)_3_] [61]. The series of symmetrical m = n system gives the expansion of both the inter- and intra-layer distances with increasing n value, while the uniaxial m ≠ n system would expand the inter- or intra-layer distance, independently, if the (*n*-C_m_H_2m+1_)_3_(*n*-C_n_H_2n+1_)N cations are located at a three-fold axis and the alkyl chain of *n*-C_n_H_2n+1_ points into the center of the hexagonal cavity surrounded by [Fe^II^Fe^III^(dto)_3_] as shown in Figure 9. Hereafter, (*n*-C_n_H_2n+1_)_4_N[Fe^II^Fe^III^(dto)_3_] as well as (*n*-C_m_H_2m+1_)_3_(*n*-C_n_H_2n+1_)N[Fe^II^Fe^III^(dto)_3_] are denoted as (*m*, *n*); e.g., (*n*-C_3_H_7_)_4_N[Fe^II^Fe^III^(dto)_3_] is (3, 3). As for (m, n), (m, n) with *m* + *n* = 8 were investigated because (4, 4) shows the bistable state of the HTP and LTP below *T*_CT_ ~ 140 K as described hereinafter, so that the electronic state of Fe(II, III) is presumably sensitive to the structural change induced by substituting the counter cation. Since single crystals of (2, 6), (3, 5), (5, 3), (6, 2), (4, 4), (5, 5), and (6, 6) were not obtained, the structural characteristics of them were investigated by the powder X-ray diffraction (PXRD) at room temperature.

Comparing the powder pattern between (3, 3) and (4, 4), both of (3, 3) and (4, 4) have the space group of *P*6_3_. In these complexes, the lattice parameters were obtained by Rietveld method. The lattice parameter, ***a***, corresponds to the intra-layer distance of Fe^II^-Fe^II^ (= Fe^III^-Fe^III^) and the lattice parameter, ***c***/2, corresponds to the inter-layer distance (≡ ***d***) of the [Fe^II^Fe^III^(dto)_3_] layer.

In the case of (5, 5), the PXRD profile cannot be refined in *P*6_3_, while it can be indexed using *P*6_3_ and trigonal *R*_3_ space groups, which implies that (5, 5) is a biphasic complex. In connection with this, many bimetallic 2D oxalato complexes exhibiting trigonal *R*3c space group have been reported [27,28,29]. In the cases of polycrystalline A[M^II^Fe^III^(ox)_3_] (A = (*n*-C_3_H_7_)_4_N, (*n*-C_4_H_9_)_4_N; M = Mn, Fe), on the other hand, the PXRD profiles show biphasic corresponding to the coexistence of *P*6_3_ and *R*3c phases [57,62,63]. In general, layered materials quite often show the structural disorder arising from the translation of rigid planes. If there are finite numbers of possible translational vectors between successive layers, (*hkl*) dependent broadening of peaks in the powder diffraction pattern arises, which is known as stacking fault. The polycrystalline (5, 5) may be also the similar case where the stacking is randomly faulted between each type, while the *R*3c phase would become a lower symmetrical group, trigonal *R*3. Since the *R*3c structure has a six-layer in a unit cell, ***a*** also gives the intra-layer nearest Fe^II^-Fe^II^ (= Fe^III^-Fe^III^) distance but ***c*** / 6 corresponds to the inter-layer distance ***d***.

In the case of polycrystalline sample of (6, 6), the similar pattern as (5, 5) is observed. This result suggests the biphasic structure of *P*6_3_ and *R*3, although only ***d*** is estimated from the (002) reflection at the lowest angle because of a lack of peaks available.

**Figure 10:**
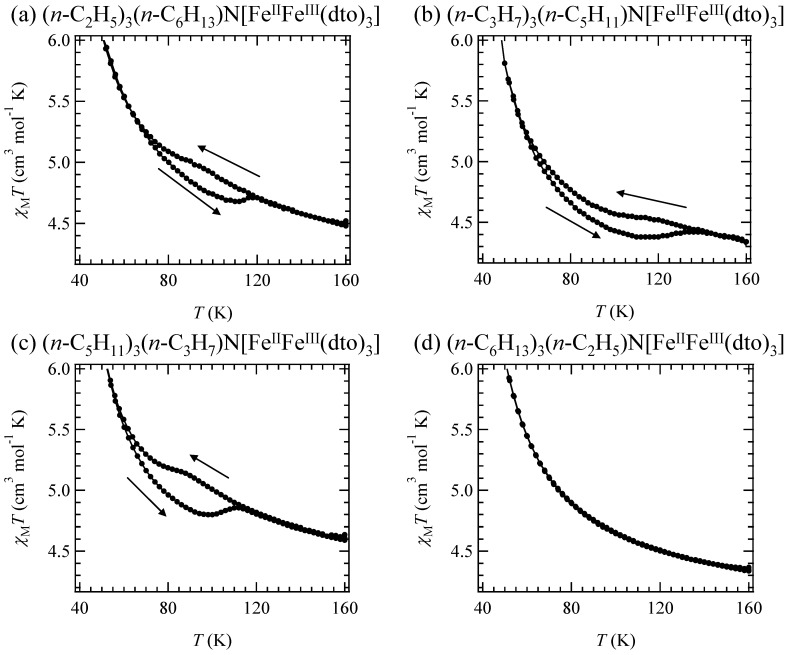
Temperature dependence of the molar magnetic susceptibility, *χ*_M_*T*, of (*n*-C_m_H_2m+1_)_3_(*n*-C_n_H_2n+1_)N[Fe^II^Fe^III^(dto)_3_](*m* + *n* = 8) on cooling and heating processes. The external magnetic field is 5000 Oe [61].

In the cases of (m, n) with m ≠ n, the PXRD profiles of (3, 5) and (5, 3) were successfully refined in *P*6_3_. The PXRD profile of (6, 2) shows the similar pattern to those of (5, 5) and (6, 6), and is assigned to the biphasic structure of *P*6_3_ and *R*3.

### 2.4. Cation size effect on the CTPT and magnetism

In the case of the uniaxial cation intercalated complexes, the effective magnetic moments at 300 K for (2,6), (3,5), (5,3) and (6,2) are 5.80, 5.64, 5.84, and 5.92 μ_B_, respectively. These values are consistent with the value from the HTP with Fe^II^(*S* = 2) and Fe^III^(*S* = 1/2). The magnetic susceptibilities for (2,6), (3,5), (5,3) and (6,2) obey the Curie-Weiss law, χ^–1^ = (*T*-θ) / *T*, in the range of 150-300 K. The Weiss constants of (2,6), (3,5), (5,3) and (6,2) are estimated at +24.1 K, +27.9 K, +24.4 K and +21.3 K, respectively. All the positive Weiss constants imply the ferromagnetic interaction between Fe^II^ and Fe^III^. In the cases of (2,6), (3,5) and (5,3), a small bump with thermal hysteresis loop appears in the χ*T* curve around 100 K, which is shown in Figure 10. This feature is quite similar to the CTPT for (3, 3) and (4, 4), which implies that the CTPT occurs in (2,6), (3,5) and (5,3). On the other hand, in the case of (6,2), the CTPT does not take place and HTP is stable even at 2 K.

**Figure 11:**
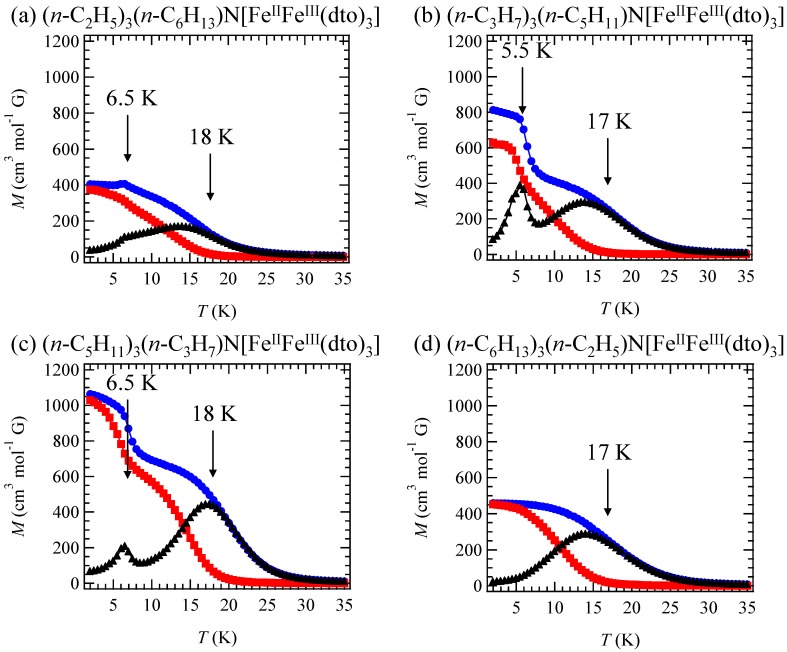
Temperature dependences of FCM, RM, and ZFCM of (*n*-C_m_H_2m+1_)_3_(*n*-C_n_H_2n+1_)N[Fe^II^Fe^III^(dto)_3_](*m* + *n* = 8). The external magnetic field is 30 Oe [61].

Figure 11 shows the FCM, RM and ZFCM for (2,6), (3,5), (5,3) and (6,2). In the cases of (2,6), (3,5) and (5,3), reflecting the coexistence of LTP and HTP even at 2 K, the LTP and HTP undergo the ferromagnetic transitions individually, which induces the stepwise ferromagnetic transition. The Curie temperatures for (2,6), (3,5), (5,3) and (6,2) are 6.5 and 18 K, 5.5 and 17 K, 6.5 and 18 K and 17 K, respectively.

The spin states of (*n*-C_m_H_2m+1_)_3_(*n*-C_n_H_2n+1_)N[Fe^II^Fe^III^(dto)_3_] with uniaxial cation are also confirmed by the ^57^Fe Mössbauer spectroscopy [61]. The ^57^Fe Mössbauer spectra of (2,6), (3,5), (5,3) and (6,2) at 300 K show the similar patterns to those of (3, 3), (4,4), (5,5) and (6, 6) where two major quadrupole doublets attributed to the Fe^II^ (*S* = 2) and Fe^III^ (*S* = 1/2) sites. In the case of (6, 2), the spectrum is almost unchanged between 200 and 77 K. On the other hand, in the cases of (2, 6), (3,5) and (5, 3), the spectra for Fe^II^(*S* = 2) and Fe^III^(*S* = 1/2) decrease and new peaks corresponding to the superposition of Fe^II^ (*S* = 0) and Fe^III^ (*S* = 5/2) appear at 77 K. This feature is quite similar to the ^57^Fe Mössbauer spectra of (3, 3) and (4, 4) at 77 K.

**Figure 12:**
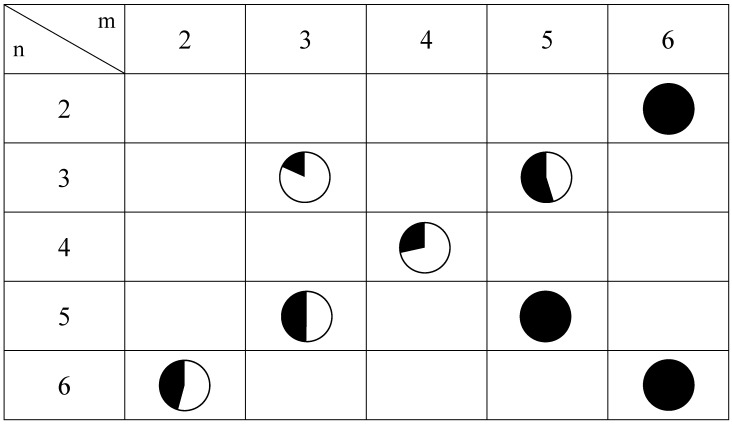
Mixing ratio between LTP and HTP for (*n*-C_m_H_2m+1_)_3_(*n*-C_n_H_2n+1_)N[Fe^II^Fe^III^(dto)_3_] obtained by the ^57^Fe Mössbauer spectroscopy at 77 K. White and black areas correspond to the LTP and HTP fractions, respectively.

From the results of the magnetic measurements and the ^57^Fe Mössbauer spectroscopy, the following feature is concluded. In the cases of (2, 6), (3,5) and(5, 3), as well as (4, 4), the CTPT occurs at around 100 K but the transition is incomplete so that the HTP and LTP coexist even at low temperature region, and consequently the individual ferromagnetic phase transitions due to the HTP and LTP are observed. On the other hand, the HTP remains in all the measuring temperature range for (6, 2) as well as the spin states for (5, 5) and (6, 6). Figure 12 shows the relationship between the spin states and the intercalated cations for (*n*-C_m_H_2m+1_)_3_(*n*-C_n_H_2n+1_)N[Fe^II^Fe^III^(dto)_3_] at 77 K, where the white and black colored areas correspond to the LTP and HTP, respectively.

**Figure 13:**
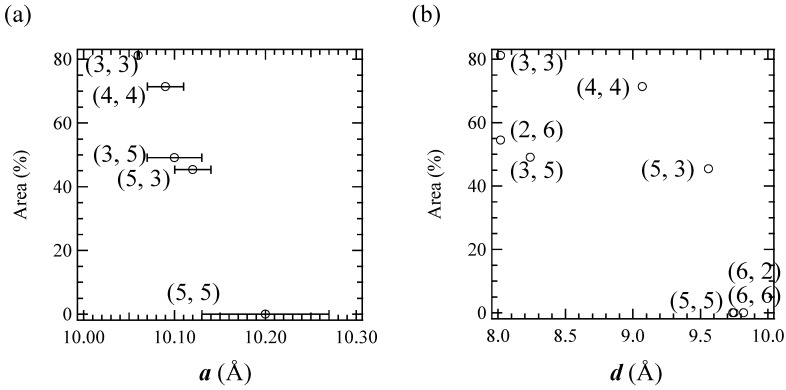
The cell parameter (**a**) ***a*** and (**b**) ***d*** dependences of the area of the ^57^Fe Mössbauer spectrum corresponding to the LTP at 77 K. In (**a**), selected (*n*-C_m_H_2m+1_)_3_(*n*-C_n_H_2n+1_)N[Fe^II^Fe^III^(dto)_3_] complexes whose ***a*** were obtained are plotted.

As shown in Figure 12, the CTPT for the (*n*-C_m_H_2m+1_)_3_(*n*-C_n_H_2n+1_)N[Fe^II^Fe^III^(dto)_3_] significantly exhibits a cation-size dependence. In order to clarify the correlation between the stability of the LTP and the crystal structure in this system, the relationship between the unit cell parameters and the area of the ^57^Fe Mössbauer spectra corresponding to the LTP at 77 K are plotted, which is shown in Figure 13. The correlation between the cell parameter ***a*** and the fraction of LTP at 77 K is shown in Figure 13(a). The fraction of the LTP tends to decrease with increasing ***a***, then becomes zero at ***a*** = 10.2±0.07 Å. On the other hand, the correlation between the cell parameter ***d*** and the fraction of LTP at 77 K is not clear. Therefore it can be concluded that the bulkier cation expands the intra-layer nearest Fe^II^-Fe^II^ (= Fe^III^-Fe^III^) distance in the 2D honeycomb network structure of [Fe^II^Fe^III^(dto)_3_], which directly stabilizes the HTP and hence the CTPT is suppressed in the system.

### 2.5. Valence fluctuation at the CTPT

As mentioned in the previous section, the charge transfer phase transition (CTPT) occurs in (*n*-C_3_H_7_)_4_N[Fe^II^Fe^III^(dto)_3_] (hereafter denoted as n = 3), while (*n*-C_5_H_11_)_4_N[Fe^II^Fe^III^(dto)_3_] (likewise, n = 5) keeps its spin state as the HTP in the whole measuring temperature range. Although the understanding of the mechanism of electron transfer in such materials is of great importance, the nature of the CTPT, dynamic properties of electron transfer in particular, has not been fully understood. The most important interest about the CTPT is whether electrons are statically localized or dynamically fluctuated between the Fe^II^ and Fe^III^ sites under thermal equilibrium condition.

To reveal the dynamics of the CTPT, muon spin relaxation (μSR) is the most powerful technique. Since the μSR is based on the observation of the evolution with time of the direction of the muon spin in the magnetic field at the muon site in the sample, it is a very useful technique that probes the magnitude, distribution, and dynamics of the internal fields. Moreover, since μSR has a wider characteristic time window (typically from 10^−5^ to 10^−11^ sec) than the ^57^Fe Mössbauer spectroscopy (around 10^−7^ sec), μSR can be a more sensitive microscopic probe to sense the dynamics of CTPT within a wider frequency range rather than ^57^Fe Mössbauer spectroscopy.

In this section, the results of zero-field (ZF) and longitudinal-field (LF) μSR measurements for n = 3 and 5 are reported to investigate the dynamics of the electron transfer between Fe^II^ and Fe^III^, which is accompanied by the CTPT.

#### 2.5.1. Zero-field muon spin relaxation

**Figure 14:**
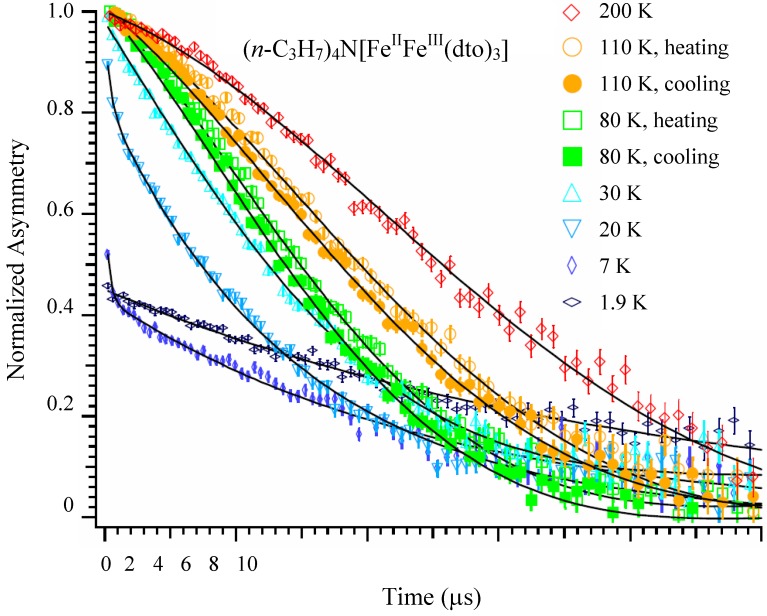
Zero-field μSR time spectra of (*n*-C_3_H_7_)_4_N[Fe^II^Fe^III^(dto)_3_]. The solid lines show the best fit of fitting function. The spectra at 80 and 110 K show both the heating and the cooling processes [41].

Figure 14 shows the ZF-μSR time spectra of n = 3 between 1.9 and 200 K [41]. A Gaussian-type depolarization behavior of the time spectrum is observed at 200 K. The time spectrum changes to an exponential type with decreasing temperature below 30 K, and a loss of the initial asymmetry is observed below 20 K. Within a time region longer than 1 μs, the time spectrum slightly recovers with decreasing temperature down to 1.9 K. This series of behaviors is a sign of the appearance of the ferromagnetically ordered state (*T*_C_ = 7 K). Furthermore, thermal hysteresis is observed around 120 K, at which the CTPT occurs. The time spectra obtained at 80 and 110 K in the cooling process are always located below those obtained at the same temperatures in the heating process. This means that the muon spin depolarizes faster in the cooling process than in the heating process. The tendency of hysteresis in the temperature dependence of the muon spin depolarization behavior around 80 K is consistent with that observed in our previous magnetic susceptibility measurement [32].

**Figure 15:**
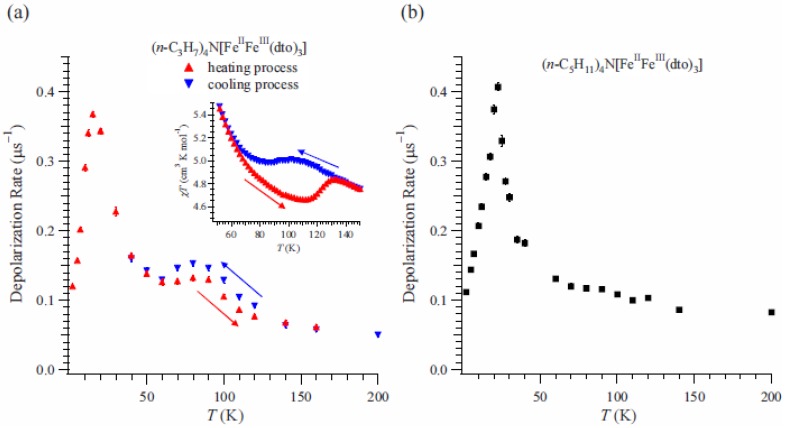
(**a**) Temperature dependence of the dynamical muon spin depolarization rate, λ_0_, for (*n*-C_3_H_7_)_4_N[Fe^II^Fe^III^(dto)_3_] on the cooling and heating processes. The inset shows the temperature dependence of the magnetic susceptibility for (*n*-C_3_H_7_)_4_N[Fe^II^Fe^III^(dto)_3_]. ← and → denote the heating and cooling processes, respectively. (**b**) Temperature dependences of λ_0_ for (*n*-C_5_H_11_)_4_N[Fe^II^Fe^III^(dto)_3_] [41].

The depolarization rate, λ_0_, of n = 3 can be obtained by fitting of these time spectra as shown in Figure 15(a) [41]. λ_0_ exhibits a peak at 15 K. Since the ferromagnetic transition temperature has been estimated at 7 K from the susceptibility measurement, the enhancement of λ_0_ above 15 K is due to the critical slowing down of the fluctuations of Fe spins toward the ferromagnetic transition [64]. Moreover, an anomalous enhancement with thermal hysteresis of λ_0_ was observed between 60 and 140 K. The thermal hysteresis of λ_0_ is presumably caused by the changes in the dynamic properties of electrons. The range of temperatures wherein the anomalous enhancement of λ_0_ was observed is well matched with the temperature range wherein the CTPT appears (see the inset of Figure 6(a)).

We also measured the ZF-μSR of n = 5 for the comparison because both the thermal hysteresis of the susceptibility and the CTPT were not observed [40]. The time spectra for n = 5 were analyzed in the same way as for n = 3. Figure 15(b) displays the temperature dependence of λ_0_ for n = 5 [41], where λ_0_ increases with decreasing temperature below 200 K. Values of λ_0_ for n = 5 are similar to those for n = 3 at the measuring temperatures except for the temperature region wherein the CTPT occurs. This fact means that depolarization mechanism of the muon spin in the case of n = 5 is similar to that of n = 3 except for the effect of CTPT. A strong enhancement was observed around 22 K, which is due to the critical slowing down of Fe spins toward the ferromagnetically ordered state like the case of n = 3. Since the anomalous enhancement of λ_0_ with thermal hysteresis around 80 K is observed only for n = 3, it is concluded that it originates from the CTPT. Taking into account that the CTPT is accompanied by the electron transfer and the HTP state is mixed with the LTP state around 80 K in the case of n = 3, it can be concluded that the motion of electrons between the Fe^II^ and Fe^III^ sites induces fluctuating internal fields at the muon site enhancing λ_0_. Therefore, the oscillation of electrons between the Fe^II^ and Fe^III^ sites is the intrinsic nature of CTPT.

#### 2.5.2. Longitudinal-field muon spin relaxation

**Figure 16:**
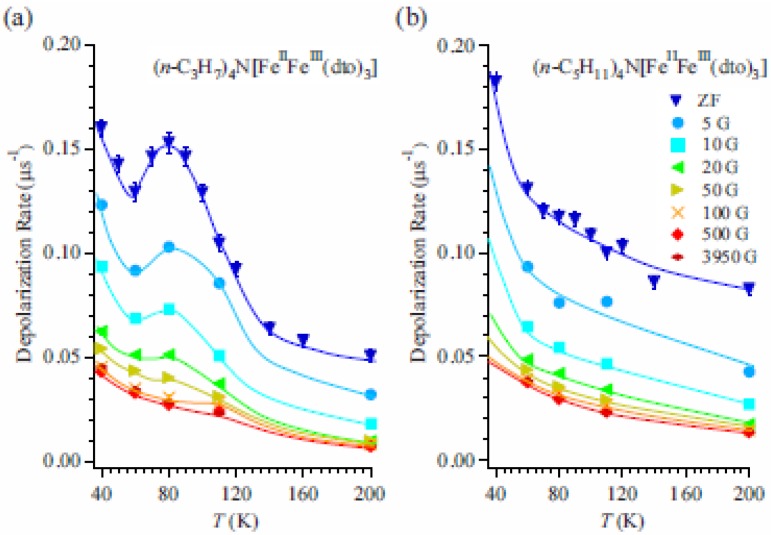
(**a**) Temperature and longitudinal-field dependence of the dynamic muon spin depolarization rate, λ_0_, for (*n*-C_3_H_7_)_4_N[Fe^II^Fe^III^(dto)_3_] and (**b**) (*n*-C_5_H_11_)_4_N[Fe^II^Fe^III^(dto)_3_] [41]. The solid lines are guides for the eye.

To obtain more detailed information on the dynamical properties of the CTPT, LF-μSR was performed at 80 K in the cooling process for both n = 3 and 5. Figures 16(a) and (b) display the temperature and LF dependences of λ_0_ for n=3 and 5, respectively. In the case of n = 3, the peak around 80 K disappears with increasing LF. λ_0_ for both n = 3 and 5 decreases with increasing LF up to about 100 Oe and becomes constant above 100 Oe. The constant values increase with decreasing temperature. These results suggest the existence of two components in the LF dependence of λ_0_. One is easily suppressed by a weak LF of about 100 Oe and shows a convex shape in its LF dependence (the weak component), and the other is independent of the LF up to 4 kOe (the strong component). The weak component means that there is a small and slowly fluctuating internal field at the muon site, which is easily masked by a small LF. Although the origin of the weak component is not clear at the moment, it would be suggested to be due to the fluctuating component of nuclear dipoles as was observed in MnSi [65]. As for the strong component, considering that the value of the strong component increases with decreasing temperature and similar values of λ_0_ are observed in both cases of n = 3 and 5 at the same temperature, it is suggested that the strong component originates from the dipole field of dynamically fluctuating Fe spins. Then, the electron transfer due to the CTPT has an additional effect on the weak component of the muon spin depolarization. This means that electrons oscillating between the Fe^II^ and Fe^III^ sites make dynamically fluctuating internal fields at the muon site.

**Figure 17:**
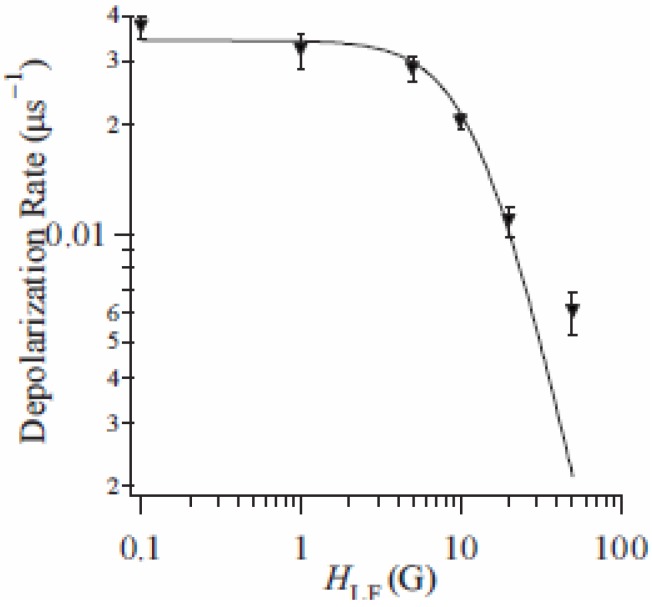
Longitudinal-field dependence of the dynamical muon spin depolarization rate, λ_CT_, for (*n*-C_3_H_7_)_4_N[Fe^II^Fe^III^(dto)_3_] at 80 K in the cooling process. The solid line shows the best fit of fitting function [41].

The weak component can be extracted from the difference of depolarization rate between n = 3 and 5. The LF dependence of this subtracted depolarization rate λ_CT_ at 80 K is summarized in Figure 17. The LF dependence of λ_CT_ shows a convex shape as a function of *H*_LF_ in a log-log plot and tends to disappear around 100 Oe. This behavior depends on the correlation time of muon spins, τ_c_, and the amplitude of the fluctuating internal field, *H*_loc_, at the muon site, respectively. τ_c_ is given as 5.7 μs from the analysis of the LF dependence of λ_CT_. The frequency of the additional internal field at the muon site, which is given as *v* = 1 / τ_c_, is on the order of 0.1 MHz at 80 K. Moreover, *H*_loc_ is estimated at 4.0 Oe, which is larger than that of contribution of the nuclear-dipole field (~1 Oe).

**Figure 18:**
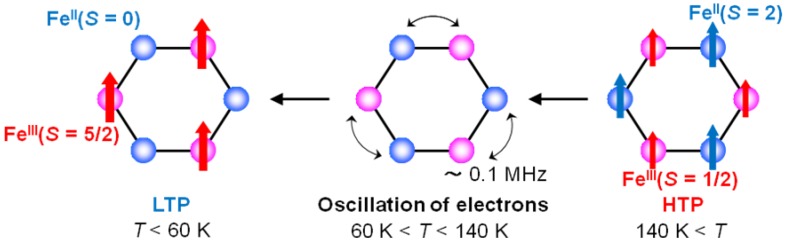
The schematic feature of the electronic oscillation in the CTPT region for (*n*-C_3_H_7_)_4_N[Fe^II^Fe^III^(dto)_3_].

In the temperature range corresponding to the CTPT, it can be suggested that the flip flop of a moving electron between Fe^II^ and Fe^III^ sites produces the dynamically fluctuating internal field at the muon site and its frequency is represented by *v*, which is schematically shown in Figure 18. The time scale of τ_c_ is consistent with the result of the ^57^Fe Mössbauer measurement, which implies that the fluctuation between the HTP and the LTP is slower than 10^−7^ s.

It was reported that the resistivity of n = 3 shows an anomalous drop with the thermal hysteresis loop within the temperature range of the CTPT [66]. Considering the present μSR results, it can be suggested that the hopping conduction in the [Fe^II^Fe^III^(dto)_3_] network is induced by the CTPT.

This result is the first observation of the dynamic electron transfer process of such mixed-valence complexes by using the μSR technique. Therefore, μSR will open the initiating research on the dynamics of charge-transfer phenomena for various inorganic and organic charge-transfer complexes that exhibit CTPT or neutral-ionic phase transitions.

### 2.6. Concerted phenomenon coupled with photoisomerization and CTPT

As mentioned in section 2.3 and 2.4, the existence of the CTPT and the ferromagnetic transition in the [Fe^II^Fe^III^(dto)_3_] system strongly depends on the size of intercalated cation. If the volume of the intercalated cation can be controlled by external stimuli, the CTPT becomes a controllable phenomenon. In organic-inorganic hybrid systems, it is effective to use an organic photochromic molecule for producing photoswitchable materials. On the basis of this strategy, we have used a photochromic spiropyran (SP) as the intercalated cation for [Fe^II^Fe^III^(dto)_3_] and have tried to control the CTPT and the ferromagnetism for (SP)[Fe^II^Fe^III^(dto)_3_] by means of photoisomerization of SP [42]. In general, the cationic spiropyran is converted from the yellow-colored closed form (CF) to the red-colored open form (OF) upon the irradiation of UV light (330-370 nm) at room temperature. The OF is usually less stable and returns to the closed form both thermally and photochemically (500-600 nm) in solution. This photoisomerization is associated with the large volume change. In the case of several kinds of spiropyran derivative, photoisomerization occurs in the solid state [67].

**Scheme 1:**
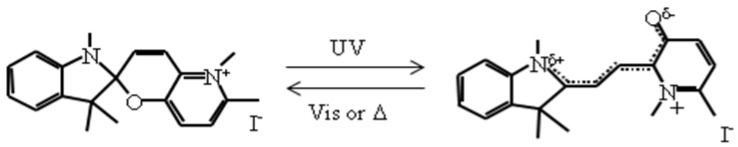
Molecular structure of spiropyran.

In this section, the photoinduced effect on the CTPT and the ferromagnetic transition of photosensitive organic-inorganic hybrid complexes, (SP-R)[Fe^II^Fe^III^(dto)_3_] (SP-R = cationic spiropyran shown in Scheme 1, R = methyl (Me), propyl (Pr)) are introduced.

#### 2.6.1.Crystal structure

**Figure 19:**
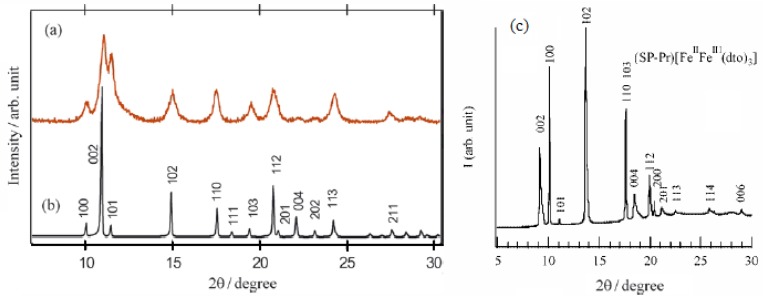
Powder X-ray diffraction profiles of (**a**) experimental diffraction profile for (*n*-C_3_H_7_)_4_N[Fe^II^Fe^III^(dto)_3_] at 300 K, (**b**) calculated one on the space group of *P*6_3_ at 300 K, and (**c**) experimental PXRD profile for (SP-Pr)[Fe^II^Fe^III^(dto)_3_] [42].

The powder X-ray diffraction patterns for (SP-Pr)[Fe^II^Fe^III^(dto)_3_] (denoted as SP-Pr complex) and (*n*-C_3_H_7_)_4_N[Fe^II^Fe^III^(dto)_3_] are shown in Figure 19 [42]. The reflection for SP-Pr complex is indexed using a hexagonal unit cell just like most dto-bridged bimetal compounds with 2D honeycomb network structure, A[M^II^M’^III^(dto)_3_] (A = (n-C_n_H_2n+1_)_4_N, (n-C_n_H_2n+1_)_4_P, M = Mn, Co, Ni, Fe, M’ = Cr, Fe). The precise crystal structure of SP-Pr complex is isostructural with (*n*-C_3_H_7_)_4_N[Fe^II^Fe^III^(dto)_3_] since the peaks over the range 10°-22° suggest a hexagonal unit cell.

**Figure 20:**
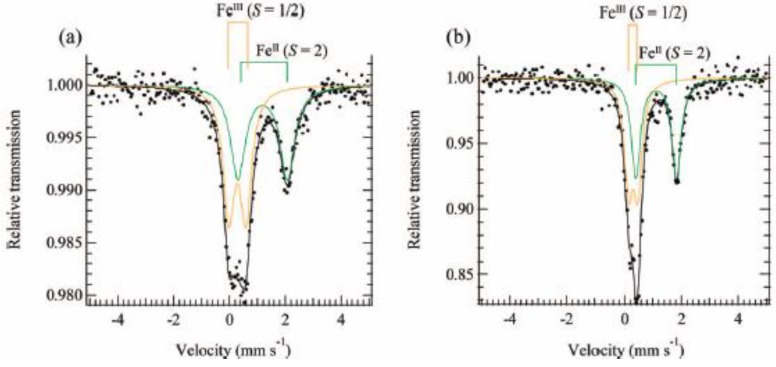
^57^Fe Mössbauer spectra of (**a**) (SP-Me)[Fe^II^Fe^III^(dto)_3_] and (**b**) (*n*-C_3_H_7_)_4_N[Fe^II^Fe^III^(dto)_3_] at room temperature [42].

The ^57^Fe Mössbauer spectra of SP-Me complex and (*n*-C_3_H_7_)_4_N[Fe^II^Fe^III^(dto)_3_] at room temperature are shown in Figure 20. The line profiles of both complexes are quite similar to each other and two quadrupole doublets are assigned to Fe^II^(*S* = 2) and Fe^III^(*S* = 1/2). The values of the isomer shifts and quadrupole splittings of SP-Me are very close to those of (*n*-C_n_H_2n+1_)_4_N[Fe^II^Fe^III^(dto)_3_] with the spin states of Fe^II^(*S* = 2) and Fe^III^(*S* = 1/2) [39]. Moreover, the infrared spectra indicate that all of the dithiooxalate groups in these complexes act as bridging ligands [68].

According to the infrared and Mössbauer spectra, the 2D honeycomb network structure of [Fe^II^Fe^III^(dto)_3_] layer is formed in SP-Me complex, where the Fe^II^(*S* = 2) and Fe^III^(*S* = 1/2) sites are coordinated by six O atoms and six S atoms, respectively.

#### 2.6.2. Photoisomerization in solid state

**Figure 21:**
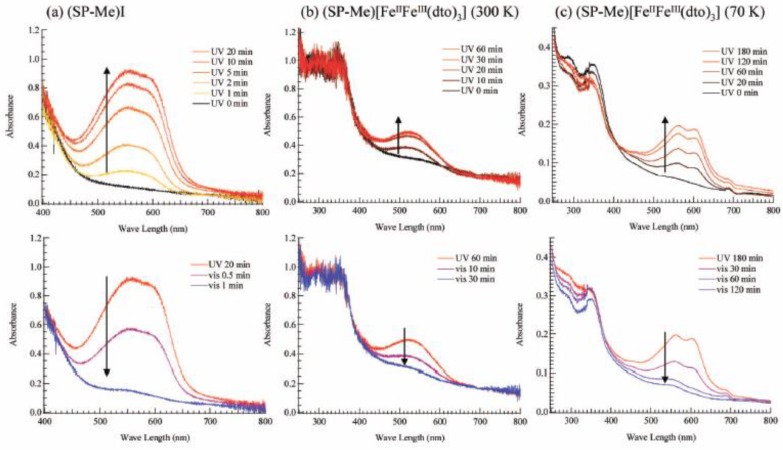
UV-vis spectral change of (**a**) (SP-Me)I and (SP-Me)[Fe^II^Fe^III^(dto)_3_] in KBr pellet at (**b**) 300 and (**c**) 70 K. UV irradiation (350 nm, 40 mW/cm^2^) on the pellet was carried out first. After the spectra were saturated, visible-light irradiation (600 mW/cm^2^) was carried out on the pellet [42].

Figure 21(a) shows the UV-vis absorption spectra for (SP-Me)I in a KBr pellet. Upon UV irradiation at 350 nm, a broad absorption band between 500 and 650 nm, corresponding to the π-π* transition of the OF, appears. In contrast, the visible light irradiation of 570 nm returned the saturated spectrum to the original one. This photochromism is based on the UV-vis light-induced equilibrium between the yellow-colored closed form (CF) and the red-colored open form (OF). Figure 21(b) and (c) show the changes of the absorption spectra for SP-Me complex in KBr pellet upon UV irradiation at 350 nm at 300 and 70 K, respectively. In the case of 300 K, the intensity of the top of the peak around 570 nm is continuously enhanced with the increase of UV irradiation time while the initial black pellet turns deep purple. After the UV irradiation for 30 min, the intensity of the absorption spectrum corresponding to the π-π* transition of the OF is almost saturated. The UV light-induced OF of SP-Me complex is stable even at room temperature in the dark condition and the purple color slowly fades to return to black in several days. In contrast, visible light irradiation considerably accelerates the color decay. This result implies that the photoisomerization of cationic SP-Me molecule from CF to OF by UV irradiation and from OF to CF by visible-light irradiation reversibly takes place in the solid state of SP-Me complex at room temperature. At 70 K, the photoisomerization of cationic SP-Me molecule is also induced by UV irradiation, and the intensity of the absorption spectrum around 570 nm is almost saturated in 180 min. In this case, the absorption band also almost disappears upon visible-light irradiation for 120 min.

#### 2.6.3. Photocontrollable CTPT and ferromagnetic transition

**Figure 22:**
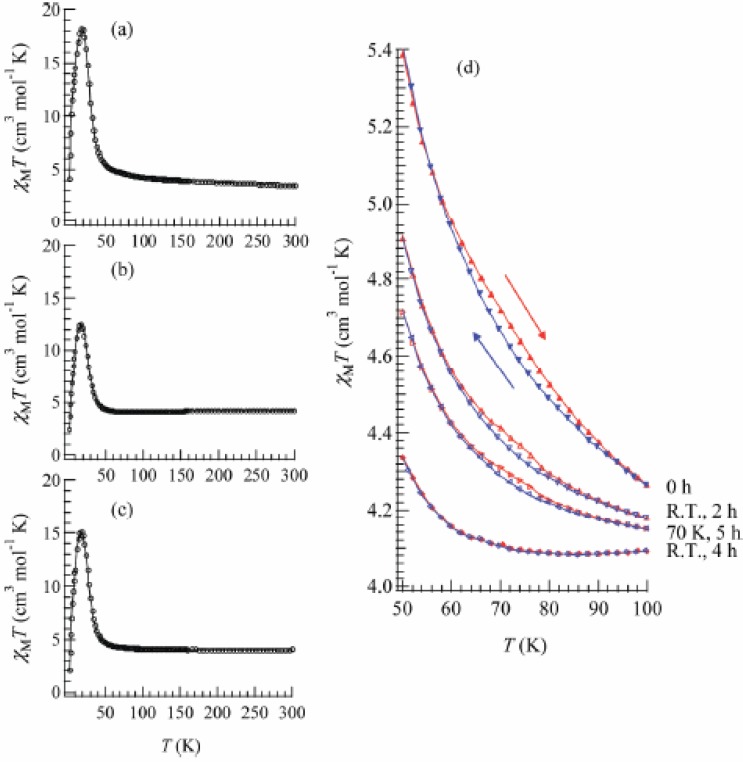
Temperature dependence of χ*T* of (SP-Me)[Fe^II^Fe^III^(dto)_3_] before (**a**) and after UV irradiation (350 nm, 40 mW/cm^2^) at (**b**) 300 K and (**c**) 70 K. Applied magnetic field was 5000 Oe. (**d**) UV irradiation effect on the χ*T versus T* in the temperature range around the charge transfer phase transition [42].

Figure 22 shows the temperature dependence of the product of the molar magnetic susceptibility and temperature, χ_M_*T* of the SP-Me complex. The effective moment at room temperature is 5.28 μ_B_ which is close to that of the spin only value of the high temperature phase (HTP) with Fe^II^ (*S* = 2) and Fe^III^ (*S* = 1/2) (5.20 μ_B_, *g* = 2). The Curie constant and Weiss constant calculated from the value above 100 K are 3.25 cm^3^·mol^-1^·K and +26.2 K, respectively. It is remarkable that the χ_M_*T* curve exhibits a thermal hysteresis loop between 50 and 100 K showing a small bump around 75 K as shown in Figure 22(d), which indicates that the CTPT occurs as well as in the case of (*n*-C_n_H_2n+1_)_4_N[Fe^II^Fe^III^(dto)_3_] (n = 3 and 4) in the similar temperature range. As the temperature is lowered below 50 K, χ_M_*T* rapidly increases up to a maximum value around 18 K and the magnetization is saturated below that temperature, which suggests that the SP-Me complex exhibits a long-range ferromagnetic ordering as well as (*n*-C_n_H_2n+1_)_4_N[Fe^II^Fe^III^(dto)_3_].

**Figure 23:**
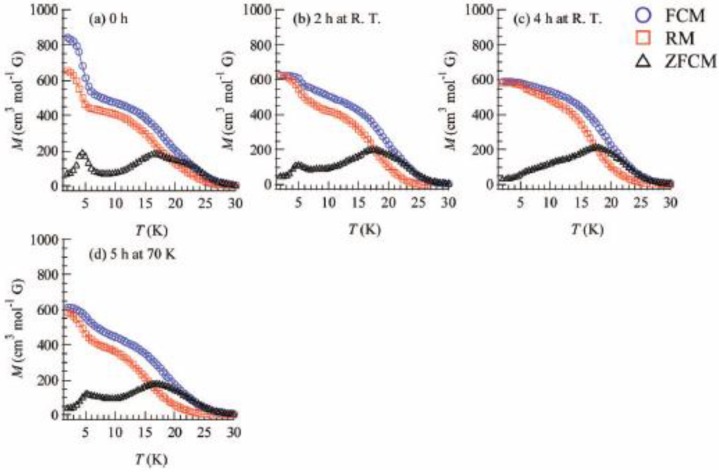
Temperature dependence of FCM, RM, and ZFCM of (SP-Me)[Fe^II^Fe^III^(dto)_3_] before **(a)** and after UV irradiation (350 nm, 40 mW/cm^2^) at 300 K (**b** and **c**) and 70 K (**d**). Applied magnetic field is 30 Oe [42].

**Figure 24:**
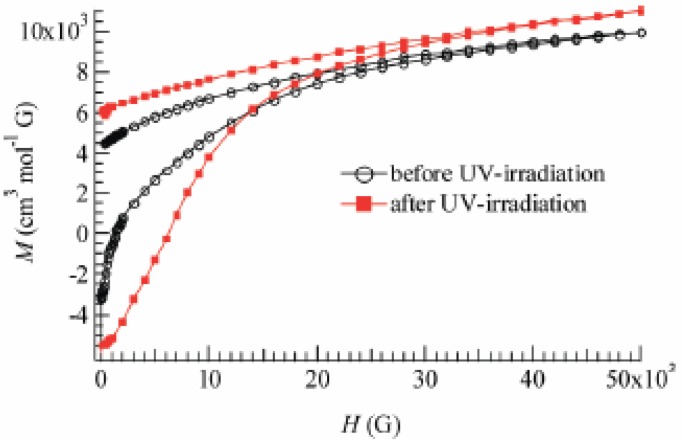
Field dependence of the magnetization of (SP-Me)[Fe^II^Fe^III^(dto)_3_] at 2 K before and after UV irradiation (350 nm, 40 mW/cm^2^) at room temperature [42].

To confirm and characterize the ferromagnetically ordered phase, the FCM, RM, ZFCM and the field dependence of the magnetization at 2 K were measured. These results are displayed in Figure 23 and Figure 24. The FCM curve decreases stepwise at 7 K and disappears at about 25 K.

As shown in Figure 23(a), the RM curve also decreases stepwise at 7 K and then disappears at about 22 K. The ZFCM curve, on the other hand, has two maxima at 5 and 18 K. This peculiar behavior of magnetization curves is quite similar to that of (*n*-C_4_H_9_)_4_N[Fe^II^Fe^III^(dto)_3_] in which the LTP and HTP coexist even in the temperature region below the CTPT. In analogy with (*n*-C_4_H_9_)_4_N[Fe^II^Fe^III^(dto)_3_], the LTP and HTP of the SP-Me complex individually undergo the ferromagnetic phase transitions with *T*_C_(LTP) = 5 K and *T*_C_(HTP) = 22 K, respectively. The field dependence of the magnetization at 2 K exhibits the hysteresis loop characteristic of ferromagnetic materials with a coercive field of 1400 Oe. The magnetization at *H* = 50000 Oe yields about 1.90 μ_B_.

Moreover, the ferromagnetism of the SP-Me complex shows a noteworthy response upon UV irradiation. Figures 23(b) and (c) show the FCM, RM, and ZFCM curves of the SP-Me complex after UV irradiation of 350 nm at room temperature. The magnetization value below 7 K starts decreasing upon the UV irradiation. The steps in the FCM and RM at 7 K are lowered, and the peak around 5 K in ZFCM disappears, indicating the disappearance of the LTP. On the other hand, the magnetization values between 7 and 30 K are slightly increased after the UV irradiation. Moreover, the thermal hysteresis loop in χ*T versus T* plot gradually vanishes with the UV irradiation, which is shown in Figure 22(d). This photo-induced effect can be explained by the suppression of the CTPT. The photoisomerization of cationic SP-Me molecule in the SP-Me complex leads to the expansion of its own volume, which gives a significant stress to the framework of [Fe^II^Fe^III^(dto)_3_] layers and expands the unit cell volume.

Taking into account that the CTPT in the (*n*-C_n_H_2n+1_)_4_N[Fe^II^Fe^III^(dto)_3_] series tends to be inhibited by the expansion of their (*n*-C_n_H_2n+1_)_4_N^+^ cation size, it can be concluded that the HTP in the SP-Me complex becomes more stable than the LTP between 2 and 300 K through the medium of the photoisomerization of cationic SP-Me molecule. In order to elucidate the mechanism of the photo-induced effect on the magnetic properties of SP-Me complex, we carried out the control experiment for the magnetism of (*n*-C_4_H_9_)_4_N[Fe^II^Fe^III^(dto)_3_] whose intercalated cation has no photochromic property. After 4 h of UV irradiation (350 nm) at room temperature, the magnetization of (*n*-C_4_H_9_)_4_N[Fe^II^Fe^III^(dto)_3_] shows no change. This result gives strong evidence that the disappearance of the LTP in the SP-Me complex after UV irradiation is caused by the photoisomerization of cationic SP-Me molecule.

**Figure 25:**
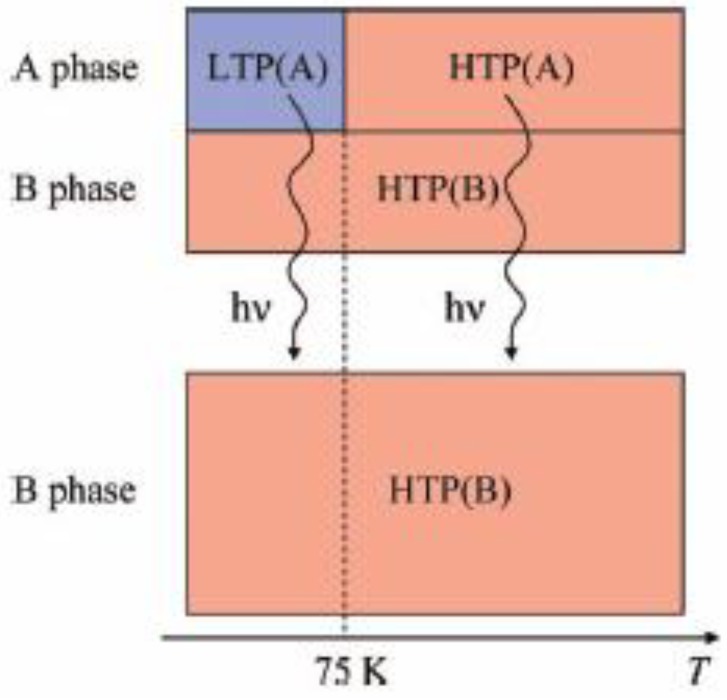
Reaction scheme of (SP-Me)[Fe^II^Fe^III^(dto)_3_] before and after UV irradiation.

**Figure 26:**
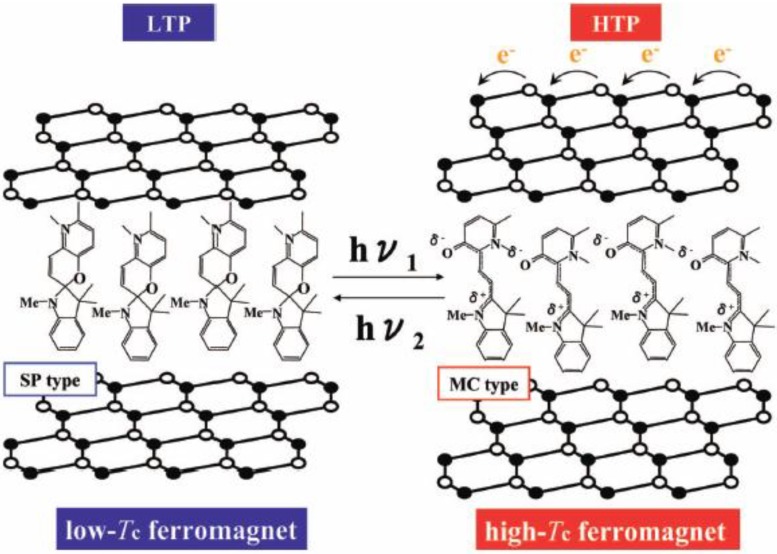
Schematic representation of the concerted phenomenon coupled with the charge transfer phase transition in [Fe^II^Fe^III^(dto)_3_] and the photoisomerization of spiropyran in (SP-Me)[Fe^II^Fe^III^(dto)_3_]. White colored and black colored circles are the Fe^II^ and Fe^III^ sites, respectively.

Figure 24 also shows the field dependence of the magnetization at 2 K after 4 h of UV irradiation at room temperature. The coercive force at 2 K is enhanced from 1400 to 6000 Oe after the UV irradiation. In connection with this, the coercive forces of (*n*-C_n_H_2n+1_)_4_N[Fe^II^Fe^III^(dto)_3_] at 2 K are 310, 3160, 6600 and 6800 Oe for n = 3, 4, 5, and 6, respectively [40], suggesting the HTP has higher coercive force than the LTP. This result supports the photo-induced HTP giving rise to long-range magnetic ordering. Before UV irradiation, the CTPT occurs around 75 K, while the LTP and HTP coexist below that temperature. There seems to be two phases in the SP-Me complex from the analysis of the temperature dependence of the magnetic susceptibility. Here we name these phases A and B respectively. The A phase undergoes the CTPT, and the HTP(A) perfectly converts into the LTP(A) with decreasing temperature. On the other hand, the B phase does not undergo the CTPT, and the HTP(B) is more stable than the LTP(B) in the whole temperature range. The reaction scheme of the SP-Me complex is illustrated in Figure 25. Suppose that the UV irradiation induces the transformation from the A phase to the B phase, the LTP(A) can be forced to convert into the HTP(B) by UV irradiation below 90 K, *i.e.*, the photoisomerization-induced CTPT schematically shown in Figure 26 is realized. Here, 90 K is the upper limit of the thermally induced CTPT in the SP-Me complex. In order to prove the concerted phenomenon coupled with the CTPT in [Fe^II^Fe^III^(dto)_3_] and the photo-isomerization of the intercalated SP-Me molecule in the SP-Me complex, we performed a low-temperature irradiation experiment which corresponds to the arrow of the left side in Figure 25. The FCM, RM, and ZFCM curves of SP-Me complex after the UV irradiation at 70 K are shown in Figure 23(d), and the χ*T* versus *T* plot of the SP-Me complex after the UV irradiation is shown in Figure 22(c). In fact, the photo-induced change in the magnetic property displays the same tendency as in the case of UV irradiation at 300 K. It should be noted that the destabilization of LTP and the stabilization of HTP under UV irradiation below 90 K induces the CTPT in the [Fe^II^Fe^III^(dto)_3_] layers. This result proves that the photoisomerization-induced CTPT is realized in this organic-inorganic hybrid system.

This new type of photomagnetism coupled with spin, charge and photon is triggered by a chemical pressure effect generated from the photoisomerization of spiropyran from the closed form to the open one in the complex. This situation seems to have significant similarity with the first events in the perception of light in rhodopsin in which photoisomerization of 11-cis-retinal into all-trans-retinal induces a conformational change in opsin and activates the associated G protein and triggers a second messenger cascade.

## 3. (*n*-C_3_H_7_)_4_N[Fe^II^Fe^III^(tto)_3_] (tto = C_2_OS_3_)

As mentioned in chapter 2, the iron mixed-valence complex (*n*-C_3_H_7_)_4_N[Fe^II^Fe^III^(dto)_3_] (dto = C_2_O_2_S_2_) shows a new-type of phase transition coupled with spin and charge around 120 K, where the charge transfer between the Fe^II^ and Fe^III^ sites occurs reversibly, and shows the ferromagnetic transition at 7 K. The research on the cation size effect and dynamic transition state for the series of the iron mixed-valence complex, [Fe^II^Fe^III^(dto)_3_], reveal the mechanism of the CTPT. Furthermore, photochromic spyropiran intercalated complex, (SP)[Fe^II^Fe^III^(dto)_3_], shows the novel photo-induced change of its spin state as an organic-inorganic hybrid material.

The magnetic state of iron mixed valence complex depends not only on the intercalated cation, but also on the bridging ligand. In the bridging ligand of oxalato derivatives, trithiooxalato (= tto (C_2_OS_3_)) and monothiooxalato (= mto (C_2_O_3_S)) ligands do not have inversion center around iron sites due to the asymmetry of these ligands. The appearance of ferroelectricity is expected from this structural character. Moreover, considering that dto bridged iron mixed-valence complexes undergo ferromagnetic transitions, (*n*-C_3_H_7_)_4_N[Fe^II^Fe^III^(tto)_3_] has a possibility to realize the coupling between ferroelectricity and ferromagnetism. In this chapter, the magnetism and the spin state of (*n*-C_3_H_7_)_4_N[Fe^II^Fe^III^(tto)_3_] are introduced.

### 3.1. CTPT and ferromagnetism

**Figure 27:**
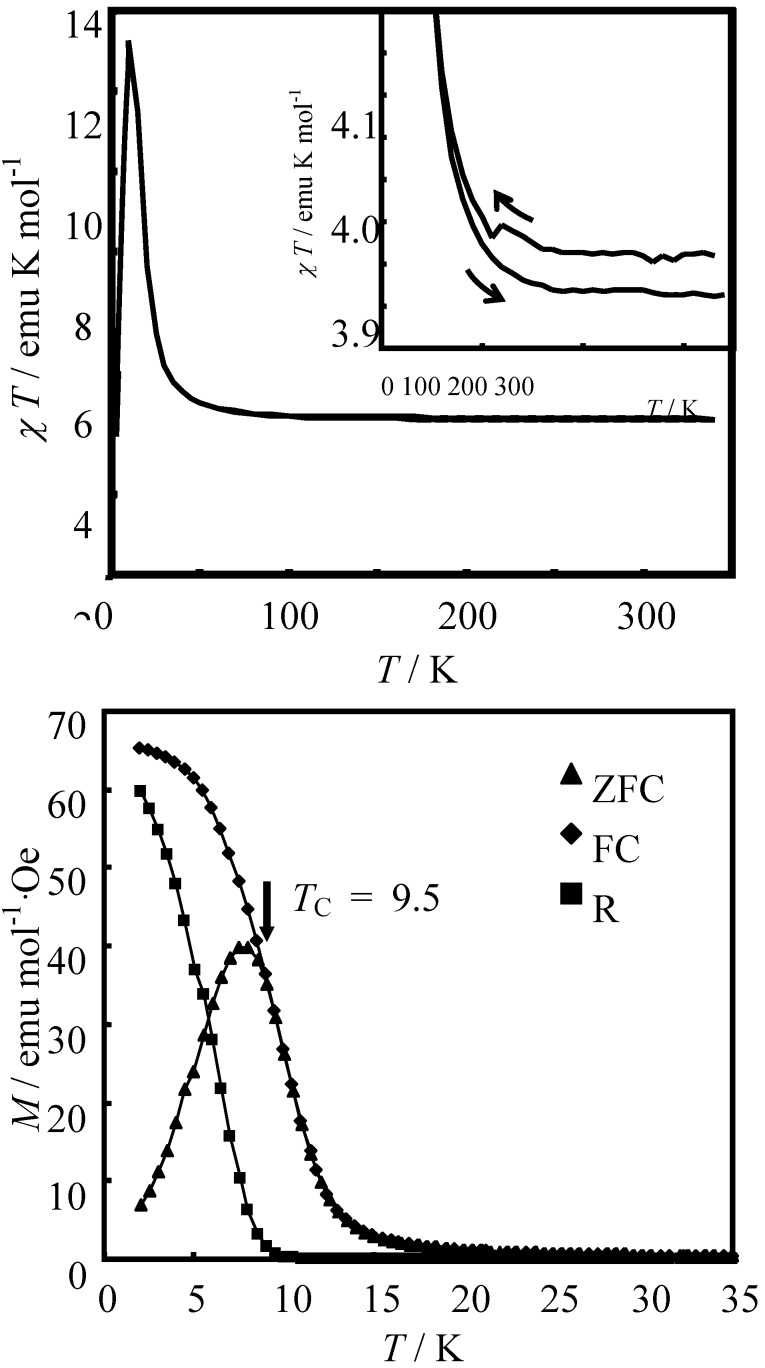
(**a**) Temperature dependence of the magnetic susceptibility multiplied by temperature (χ*T*) for (*n*-C_3_H_7_)_4_N[Fe^II^Fe^III^(tto)_3_]. Inset shows on enlarged view of χ*T* plot to emphasize the hysteresis behavior. (**b**) ZFC, FC and RM plots. *T*_C_ is denoted by an arrow [69].

As shown in Figure 27(a), (*n*-C_3_H_7_)_4_N[Fe^II^Fe^III^(tto)_3_] shows a hysteresis in the temperature dependence of χ*T* [69]. The lower end of this hysteresis is estimated at 50 K, and the higher end of the hysteresis cannot be observed up to 340 K. The difference between the cooling and heating process of χ*T* plot is reproducible on repeated runs. When the heating process returns to cooling process at 340 K, χ*T* value jumps from 3.86 emu·K·mol^-1^ to 3.91 emu·K·mol^-1^ immediately (The dotted arrow in inset of Figure 27(a) schematically shows this behavior). This jump can be observed in the cycle via 300 K (see dotted arrow in inset of Figure 27(a)) therefore, this jump does not necessarily represent the higher end of the hysteresis. In the case of (*n*-C_3_H_7_)_4_N[Fe^II^Fe^III^(dto)_3_], this type of hysteresis loop, which is caused by the CTPT, is observed in temperature range between 60-125 K as shown in the section 2.2. On the other hand, both ends of hysteresis is stretched from 50 K to over 340 K in (*n*-C_3_H_7_)_4_N[Fe^II^Fe^III^(tto)_3_]. This result shows that the transition temperature between the HTP with the Fe^III^(*S* = 1/2) and Fe^II^(*S* = 2) states and the LTP with the Fe^III^(*S* = 5/2) and Fe^II^(*S* = 0) crosses room temperature. Figure 27(b) shows the ZFC, FC and RM behaviors below 35 K. Judging from these results, (*n*-C_3_H_7_)_4_N[Fe^II^Fe^III^(tto)_3_] undergoes a ferromagnetic transition with *T*_C_ = 9.5 K.

**Figure 28:**
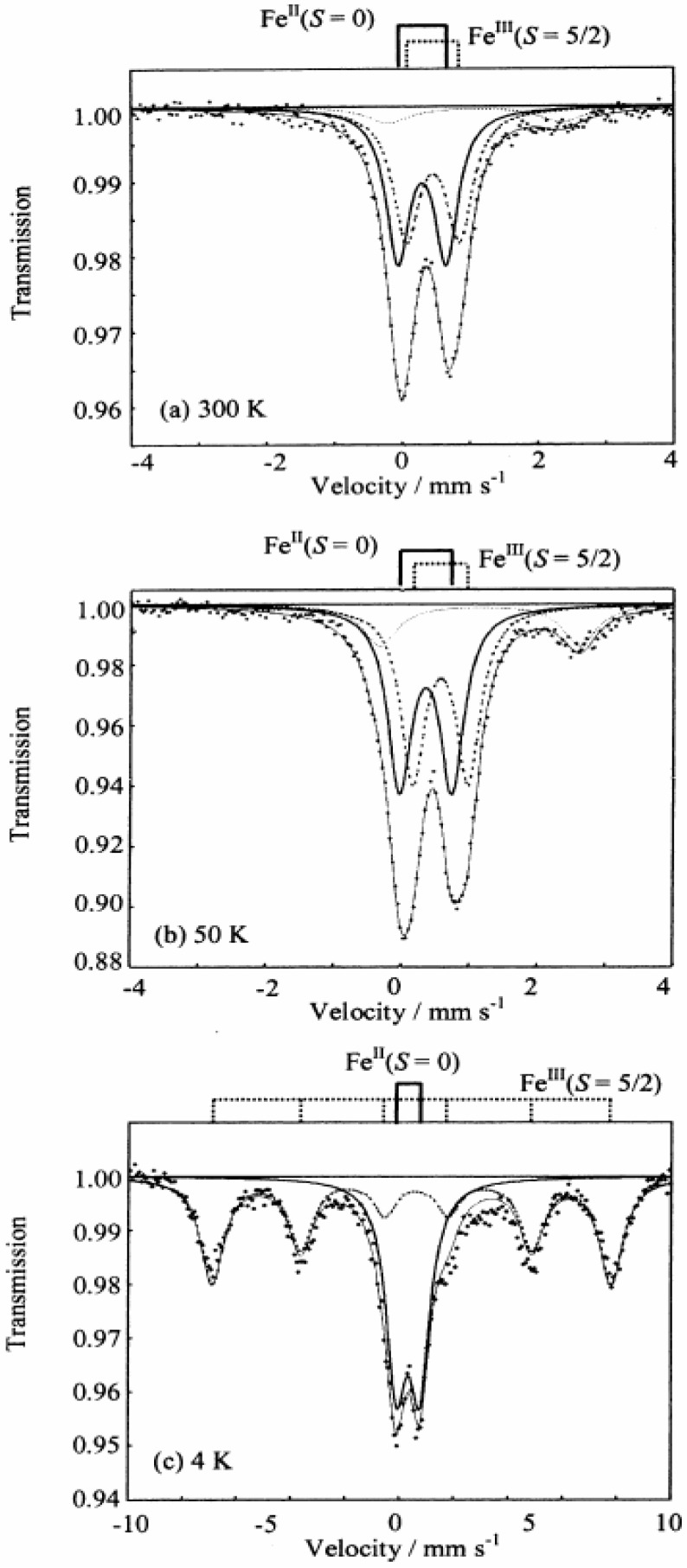
^57^Fe Mössbauer spectra of (*n*-C_3_H_7_)_4_N[Fe^II^Fe^III^(tto)_3_] at (**a**) 300 K, (**b**) 50 K and (**c**) 4 K. Solid and dashed lines denote the Mössbauer profiles of Fe^II^(*S* = 0) and Fe^III^ (*S* = 5/2), respectively. Dotted lines in (a) and (b) is assigned to Fe^II^(*S* = 2) as a minor component [69].

Figure 28 shows the ^57^Fe Mössbauer spectra for (*n*-C_3_H_7_)_4_N[Fe^II^Fe^III^(tto)_3_]. The line profiles at 300 K and 50 K are almost same. Figure 28(c) obviously shows the hyperfine splitting caused by internal magnetic field. Judging from the line shape of Figure 28(c), the ^57^Fe Mössbauer spectrum with six branches induced by internal magnetic field is attributed to that for the Fe^III^(*S* = 5/2) site. The doublet peak with isomer shift (*IS*) of 0.34 mm/s and quadrupole splitting (*QS*) of 0.87 mm/s without internal magnetic field is attributed to that for the Fe^II^(*S* = 0) site. Taking account of the Mössbauer spectrum at 4 K and the magnitude of magnetization estimated by the magnetic susceptibility measurement, the line profiles at 300 K and 50 K are assigned to the Fe^III^(*S* = 5/2) and Fe^II^(*S* = 0) sites. The Mössbauer parameters are quite similar to those of the LTP of (*n*-C_3_H_7_)_4_N[Fe^II^Fe^III^(dto)_3_]. Additionally, minor fractions with *IS* ≈ 1.1 mm/s and *QS* ≈ 2.7 mm/s are found in Figures 28(a) and (b). Since these *IS* and *QS* values are characteristics of Fe^II^(*S* = 2), this fraction belongs to the HTP with the Fe^III^(*S* = 1/2) and Fe^II^(*S* = 2) states. Therefore, (*n*-C_3_H_7_)_4_N[Fe^II^Fe^III^(tto)_3_] mainly shows the LTP with Fe^III^(*S* = 5/2) and Fe^II^(*S* = 0) states, while it contains the fraction of HTP with the Fe^III^(*S* = 1/2) and Fe^II^(*S* = 2) states.

**Figure 29:**
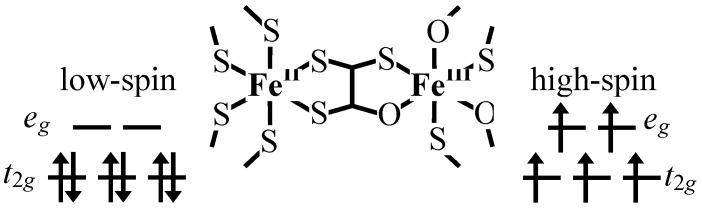
The electronic state for as grown sample of (*n*-C_3_H_7_)_4_N[Fe^II^Fe^III^(tto)_3_] at 300 K.

These results suggest that the CTPT between the HTP and LTP is realized for (*n*-C_3_H_7_)_4_N[Fe^II^Fe^III^(tto)_3_] around room temperature. Judging from the ^57^Fe Mössbauer spectra on heating process, the spin state of (*n*-C_3_H_7_)_4_N[Fe^II^Fe^III^(tto)_3_] is essentially consistent with the LTP with Fe^II^(S = 0) and Fe^III^(S = 5/2), which is schematically shown in Figure 29.

## 4. (*n*-C_n_H_2n+1_)_4_N[Fe^II^Fe^III^(mto)_3_] (mto = C_2_O_3_S)

In general, the Fe site coordinated by six S atoms is in the low spin state, while the Fe site coordinated by six O atoms is in the high spin state. From this viewpoint, we have synthesized an monothiooxalato-bridged iron mixed-valence complexes, (*n*-C_4_H_9_)_4_N[Fe^II^Fe^III^(mto)_3_], consisting of Fe^III^O_3_S_3_ and Fe^II^O_6_ octahedra [53], In this system, the spin state of Fe^III^ is considered to be situated in the spin crossover region between the low-spin state of *S* = 1/2 and the high-spin state of *S* = 5/2.

### 4.1. Ferrimagnetism

**Figure 30:**
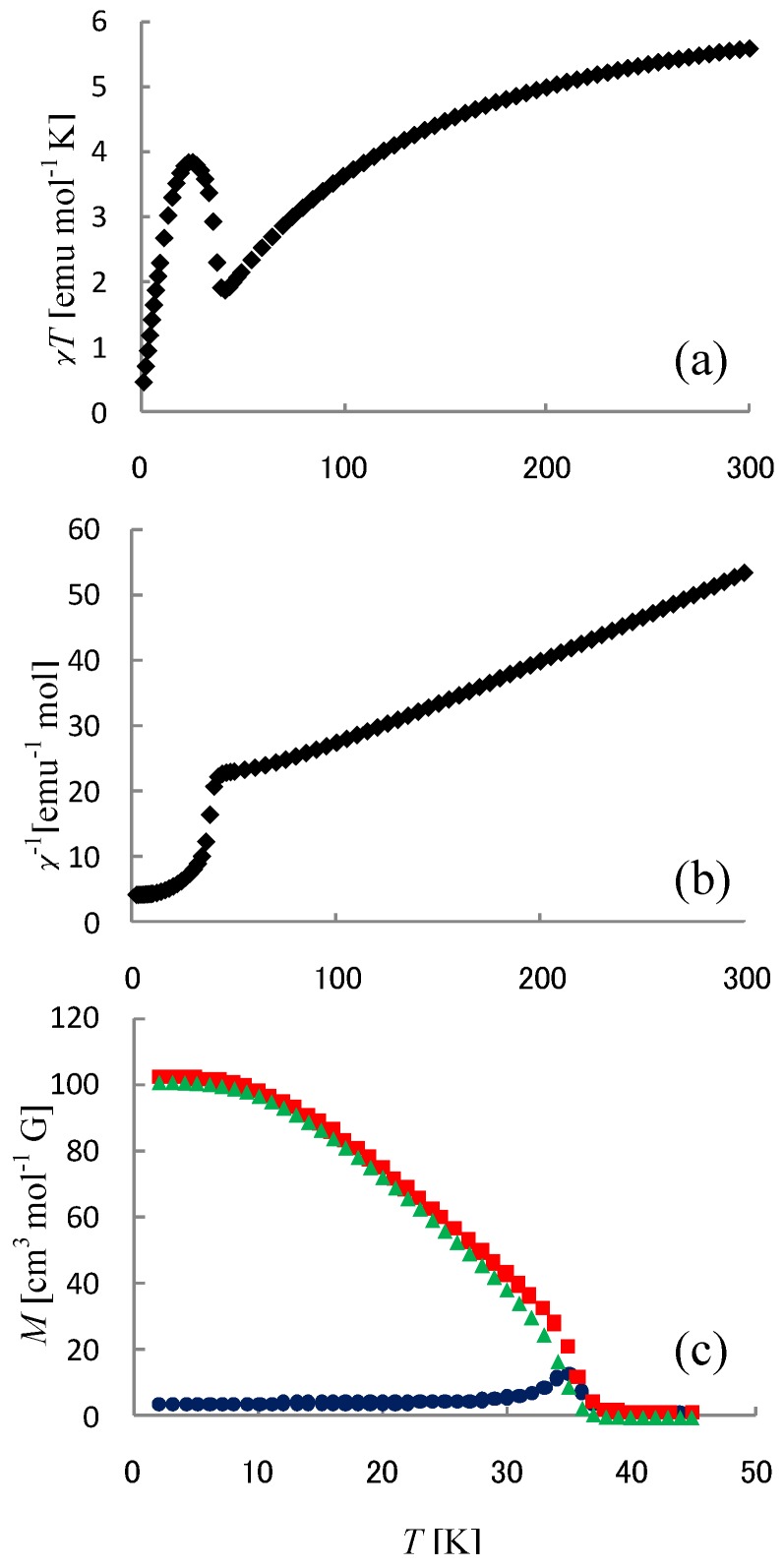
Temperature dependences of (a) effective magnetic moment, (b) inverse magnetic susceptibility and (c) magnetization curves (■: FCM, ▲: RM, ●: ZFCM) for (*n*-C_4_H_9_)_4_N[Fe^II^Fe^III^(mto)_3_] [53].

Figure 30(a) shows the molar magnetic susceptibility as a function of temperature, *χ*_M_*T*, for (*n*-C_4_H_9_)_4_N[Fe^II^Fe^III^(mto)_3_]. The effective magnetic moment, *μ_eff_*, decreases with decreasing temperature down to the minimum 1.87 μ_B_ at 42 K, and it increases abruptly up to the maximum of 3.85 μ_B_ at 26 K, then decreases again. This behavior is typical of ferrimagnetism. The effective moment at room temperature is 5.60 μ_B_. The spin-only magnetic moment is 7.68 μ_B_ for the combination of Fe^III^(*S* = 5/2) and Fe^II^(*S* = 2). On the other hand, that is 5.19 μ_B_ for the combination of Fe^III^(*S* = 1/2) and Fe^II^(*S* = 2). Therefore, the effective magnetic moment of Fe^III^ in (*n*-C_4_H_9_)_4_N[Fe^II^Fe^III^(mto)_3_] is situated in the middle value between the magnetic moments for the high-spin state (*S* = 5/2) and the low-spin one (*S* = 1/2). This implies the possibility that the spin state of Fe^III^ is the spin equilibrium between the high spin and the low spin states.

The inverse magnetic susceptibility as a function of temperature is shown in Figure 30(b). From the fitting of inverse magnetic susceptibility with the Curie-Weiss law, *χ*^-1^ = (*T* – *θ* ) / *C*, Weiss constant, *θ*, is estimated at – 93 K. In order to confirm the ferrimagnetic phase-transition, FCM, ZFCM and RM were investigated. This result is shown in Figure 30(c). From the analysis of magnetization curves, the ferrimagnetic transition temperature is estimated at 38 K. The magnetic property of (*n*-C_4_H_9_)_4_N[Fe^II^Fe^III^(mto)_3_] is similar to that of (*n*-C_4_H_9_)_4_N[Fe^II^Fe^III^(ox)_3_] which shows the ferrimagnetic phase-transition at *T*_N_ = 43 K [70].

### 4.2. Iron valence fluctuation

**Figure 31:**
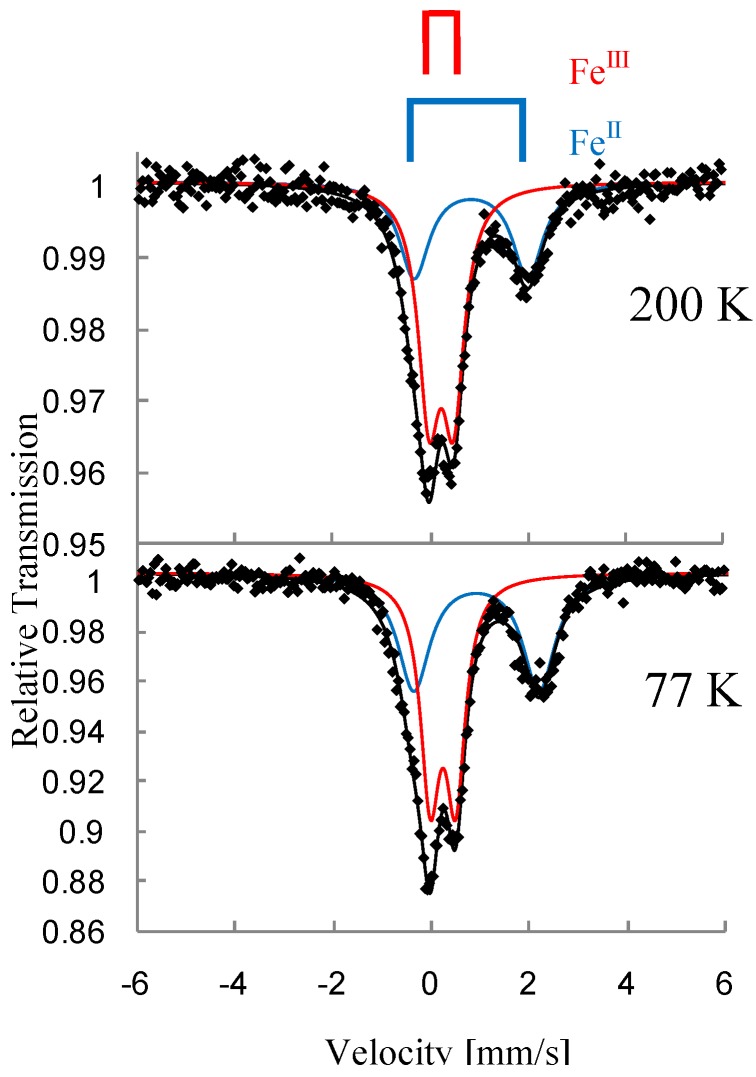
^57^Fe Mössbauer spectra of (*n*-C_4_H_9_)_4_N[^57^Fe^II^Fe^III^(mto)_3_] at 200 and 77 K [53].

**Figure 32:**
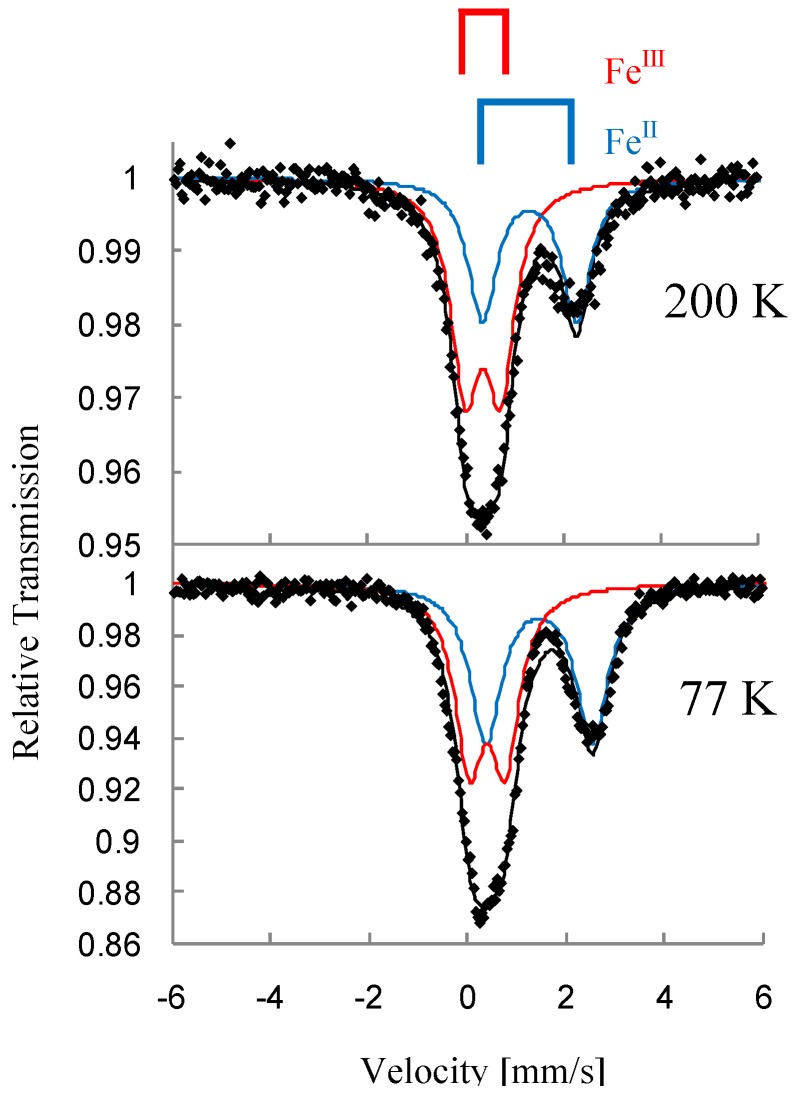
^57^Fe Mössbauer spectra of (*n*-C_4_H_9_)_4_N[Fe^II 57^Fe^III^(mto)_3_] at 200 and 77 K [53].

Figure 31 and Figure 32 show the ^57^Fe Mössbauer spectra of (*n*-C_4_H_9_)_4_N[^57^Fe^II^Fe^III^(mto)_3_] and (*n*-C_4_H_9_)_4_N[Fe^II^^57^Fe^III^(mto)_3_] at 200 and 77 K, respectively. In both of the ^57^Fe Mössbauer spectra of (*n*-C_4_H_9_)_4_N[^57^Fe^II^Fe^III^(mto)_3_] and (*n*-C_4_H_9_)_4_N[Fe^II^^57^Fe^III^(mto)_3_], the spectra clearly indicate the presence of two quadrupole doublets assigned to Fe^II^ (*S* = 2) and Fe^III^ (*S* = 5/2).

The spectra of ^57^Fe coordinated by O_6_ is observable for (*n*-C_4_H_9_)_4_N[^57^Fe^II^Fe^III^(mto)_3_], while that of ^57^Fe coordinated by S_3_O_3_ is observable in (*n*-C_4_H_9_)_4_N[Fe^II^^57^Fe^III^(mto)_3_]. The value of the quadrupole splitting (*QS*) of the Fe^III^ site coordinated by S_3_O_3_ is larger than that of the Fe^III^ site coordinated by O_6_, which implies that the Fe^III^ site coordinated by S_3_O_3_ is in the highly distorted octahedral environment. Although only one Fe site was enriched with ^57^Fe isotope in preparation, the observed spectra show a considerable amount of the other Fe site, which suggests that the charge transfer between Fe^II^ and Fe^III^ partially occurs. The time scale of the charge transfer between Fe^II^ and Fe^III^ is slower than the time window of the ^57^Fe Mössbauer spectroscopy (10^-7^ s).

**Figure 33:**
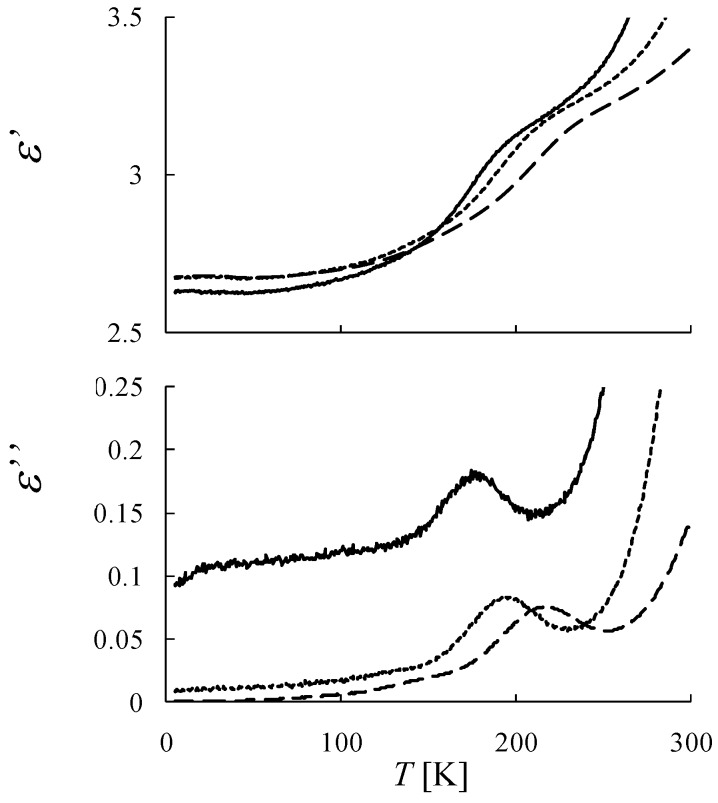
Temperature dependence of the dielectric constants for (*n*-C_4_H_9_)_4_N[Fe^II^Fe^III^(mto)_3_], solid lines: 1 kHz, dotted lines: 10 kHz, dashed lines: 100 kHz. [53].

Figure 33 shows the result of the dielectric constant measurements. As shown in Figure 33, anomalous enhancements in the real and imaginary parts of the dielectric constant appear above *T*_N_. The anomalous enhancements depend on the frequency of the alternating electric field. In the imaginary part of the dielectric constant, the peak position of the anomalous enhancement shifts to the higher temperature side with increasing the frequency of the alternating electric field. In the dielectric measurements of (*n*-C_3_H_7_)_4_N[Fe^II^Fe^III^(dto)_3_], the anomalous enhancements attributed to the charge transfer phase transition are observed at 120 K with thermal hysteresis and is independent of the frequency of alternating electric field. As mentioned above, it should be considered that the anomalous enhancement in the dielectric constant of (*n*-C_4_H_9_)_4_N[Fe^II^Fe^III^(mto)_3_] corresponding to the valence fluctuation between Fe^II^ and Fe^III^ whose time scale depends on temperature. From the analysis of the ^57^Fe Mössbauer spectra and the dielectric constant, the time scale of the charge transfer between Fe^II^ and Fe^III^ is slower than 10^-7^ s. From the fact that the anomalous enhancement in the dielectric constant of (*n*-C_3_H_7_)_4_N[Fe^II^Fe^III^(dto)_3_] appears only at the charge transfer phase transition, while that of (*n*-C_4_H_9_)_4_N[Fe^II^Fe^III^(mto)_3_] appears in a wide temperature range, it should be considered that the charge transfer of (*n*-C_4_H_9_)_4_N[Fe^II^Fe^III^(mto)_3_] is the valence fluctuation without phase transition.

### 4.3. Rapid spin equilibrium and its effect on the iron valence fluctuation

(*n*-C_n_H_2n+1_)_4_N[Fe^II^Fe^III^(mto)_3_] (mto = C_2_O_3_S) consists of Fe^III^O_3_S_3_ and Fe^II^O_6_ octahedra. In this system, the spin state of Fe^II^O_6_ is the high-spin state of *S* = 2, while that of Fe^III^O_3_S_3_ is considered to be situated in the spin crossover region between the low-spin state of *S* = 1/2 and the high-spin state of *S* = 5/2, which is strongly supported by the fact that the Fe^III^O_3_S_3_ sites in tris(monothio-β-diketonato)iron(III) complexes [71] and tris(monothiocarbamato)iron(III) complexes [72] exhibit spin equilibrium phenomena.

**Figure 34:**
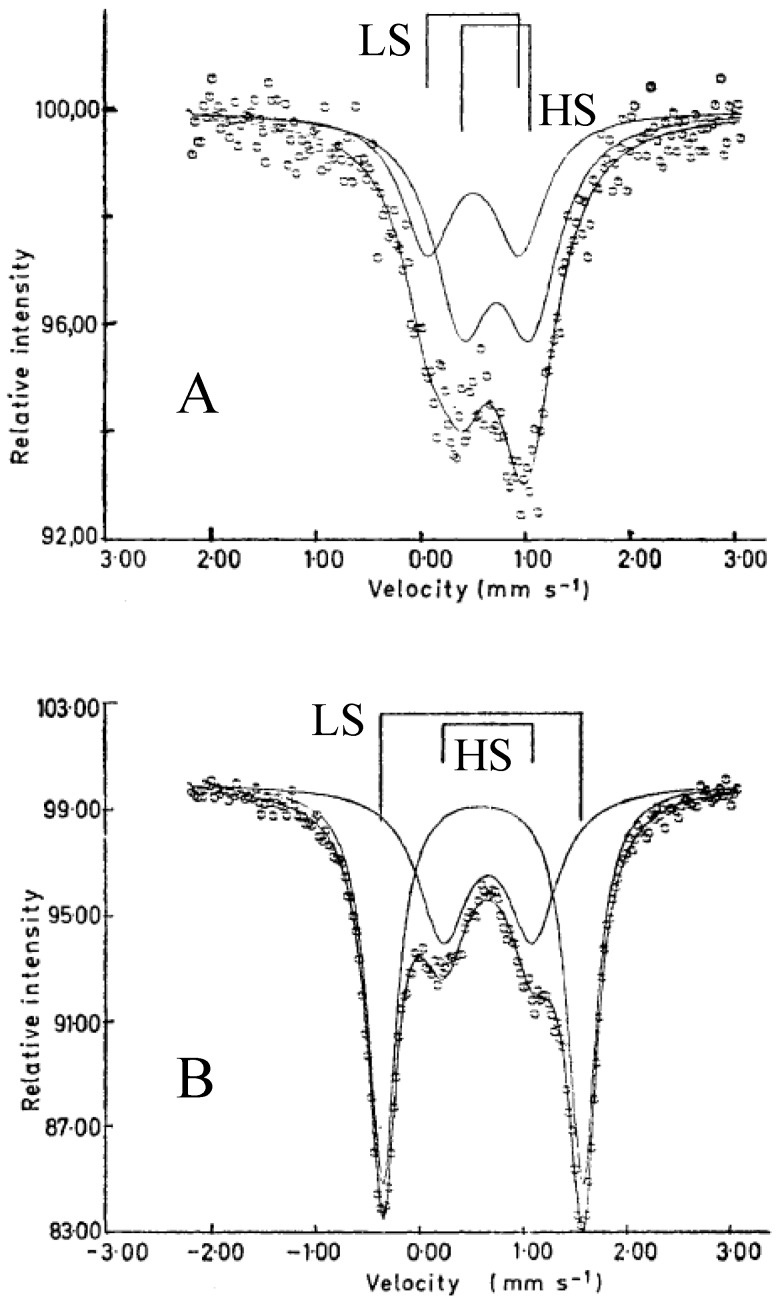
^57^Fe Mössbauer spectra of tris(monothio-β-diketonato)iron(III) complex at (A) 300 K and (B) 80 K. [71].

**Figure 35:**
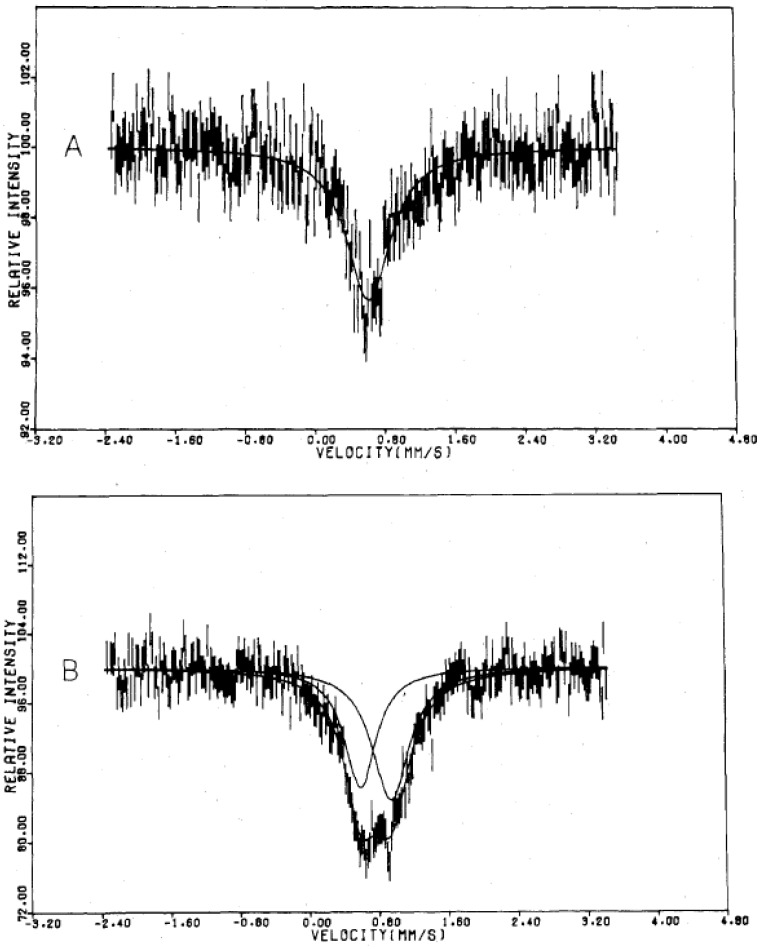
^57^Fe Mössbauer spectra of tris(monothiocarbamato)iron(III) complex, [Fe(III)(Et_2_mtc)_3_] at (A) 296 K and (B) 93 K. [72].

In connection with this, the following should be mentioned. In the case of tris(monothio-β-diketonato)iron(III) complexes, the low-spin and high-spin states coexist in the whole measuring temperature between 300 K and 80 K, and two kinds of doublet corresponding to the low-spin and high-spin states are clearly distinguished in the ^57^Fe Mössbauer spectra (Figure 34), where the area of the low-spin state increases with decreasing temperature from 300 K to 80 K [71]. In the case of tris(monothiocarbamato)iron(III) complexes, on the other hand, the rapid spin equilibrium occurs in which the high-spin and low-spin states exchange in the time scale of less than 10^-7^ s. In this case, the averaged single doublet between the high-spin state (*S* = 5/2) and low-spin (*S* = 1/2) state is observed, which is shown in Figure 35 [72].

**Figure 36:**
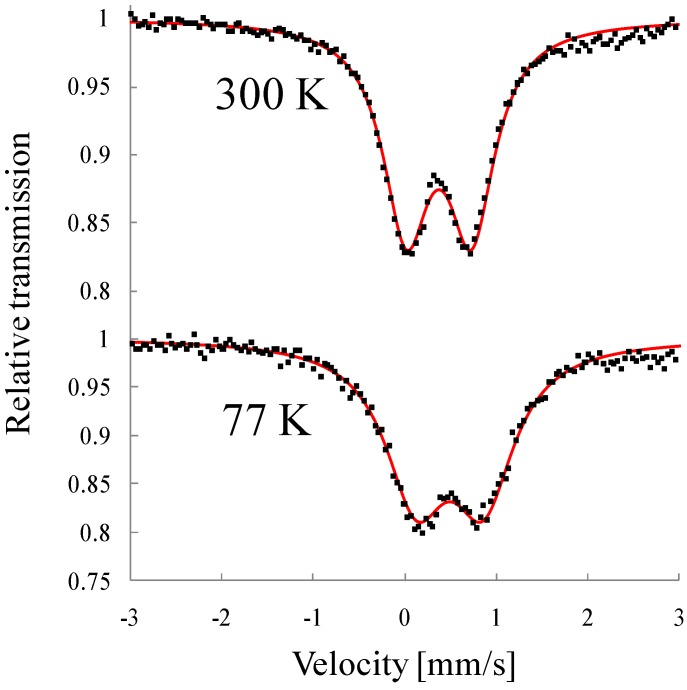
^57^Fe Mössbauer spectra of (*n*-C_3_H_7_)_4_N[Cd^II^Fe^III^(mto)_3_] at 300 K and 77 K.

In the case of (*n*-C_4_H_9_)_4_N[Fe^II^Fe^III^(mto)_3_], the effective magnetic moment is situated in the middle value between the magnetic moment for the high-spin and the low-spin states of Fe^III^. This implies that the spin state of Fe^III^ is the spin equilibrium of the high spin state of *S* = 5/2 and the low spin state of *S* = 1/2, which is supported by the coexistence of ESR signals corresponding to the high spin state and the low spin state [73]. In the ^57^Fe Mössbauer spectra of (*n*-C_4_H_9_)_4_N[Fe^II^^57^Fe^III^(mto)_3_], the presence of two quadrupole doublets consisting of Fe^II^O_3_S_3_ (*S* = 2) and the averaged spectrum of Fe^III^O_3_S_3_ between the high-spin state (S = 5/2) and low-spin state (*S* = 1/2) in the whole measuring temperature between 300 K and 5 K. The averaged single doublet at the Fe^III^S_3_O_3_ site between the high-spin state (*S* = 5/2) and low-spin (*S* = 1/2) state in the whole measuring temperature between 300 K and 5 K is quite similar to those of tris(monothiocarbamato)iron(III) complexes exhibiting rapid spin equilibrium phenomena. From the analysis of the ^57^Fe Mössbauer spectra, the magnetic susceptibility, and ESR spectra, it is concluded that the rapid spin equilibrium in which the high-spin state and low-spin state exchange in the time scale of less than 10^-7^ s occurs at the Fe^III^ site coordinated by three O atoms and three S atoms in (*n*-C_4_H_9_)_4_N[Fe^II^Fe^III^(mto)_3_]. Figure 36 shows the ^57^Fe Mössbauer spectra for (*n*-C_3_H_7_)_4_N[Cd^II^Fe^III^(mto)_3_] at 300 K and 77 K, whose averaged single doublet between the high-spin state (*S* = 5/2) and low-spin (*S* = 1/2) state strongly supports that the spin state of Fe^III^O_3_S_3_ in (*n*-C_4_H_9_)_4_N[Fe^II^Fe^III^(mto)_3_] is in the rapid spin equilibrium between the low-spin state of *S* = 1/2 and the high-spin state of *S* = 5/2.

**Figure 37:**
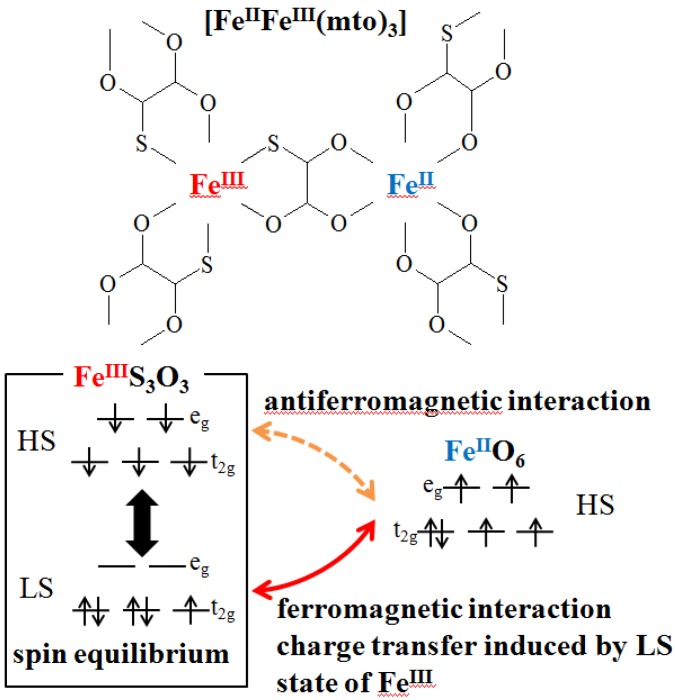
Schematic feature of the rapid spin equilibrium in the Fe^III^O_3_S_3_ site and its effect on the iron valence fluctuation in (*n*-C_4_H_9_)_4_N[Fe^II^Fe^III^(mto)_3_].

Finally, we discuss the rapid spin equilibrium and its effect on the iron valence fluctuation. As mentined above, in (*n*-C_4_H_9_)_4_N[Fe^II^Fe^III^(mto)_3_], the spin state of the Fe^II^O_6_ site is the high spin state of *S* = 2, while that of the Fe^III^S_3_O_3_ site exhibits a rapid spin equilibrium between the high-spin state and low-spin state in the time scale of less than 10^-7^ s, which is schematically shown in Figure 37. On the analogy of (*n*-C_4_H_9_)_4_N[Fe^II^Fe^III^(ox)_3_] which shows the ferrimagnetic phase-transition at *T*_N_ = 43 K [70], the magnetic interaction between the high spin state (*S* = 5/2) of the Fe^III^S_3_O_3_ site and the high spin state (*S* = 2) of the Fe^II^O_6_ site should be antiferromagnetic, which is responsible for the ferromagnetic transition at 38 K. On the other hand, the magnetic interaction between the low spin state (*S* = 1/2) of the Fe^III^S_3_O_3_ site and the high spin state (*S* = 2) of the Fe^II^O_6_ site should be ferromagnetic, on the analogy of (*n*-C_3_H_7_)_4_N[Fe^II^Fe^III^(dto)_3_] which undergoes the ferromagnetic transition at *T*_C_ = 7 K. The ferromagnetic coupling between the low spin state (*S* = 1/2) of the Fe^III^S_3_O_3_ site and the high spin state (S = 2) of the Fe^II^O_6_ site induces the electron transfer between the *t*_2g_ orbitals of the Fe^II^ and Fe^III^ sites. However, in the case of the antiferromagnetic interaction between the high spin state (*S* = 5/2) of the Fe^III^S_3_O_3_ site and the high spin state (*S* = 2) of the Fe^II^O_6_ site, any electron transfer is forbidden because of the breakdown of Hund’s rule. In this way, the rapid spin equilibrium of the Fe^III^S_3_O_3_ site induces the valence fluctuation of the FeS_3_O_3_ and FeO_6_ sites in (*n*-C_4_H_9_)_4_N[Fe^II^Fe^III^(mto)_3_] through the ferromagnetic coupling between the low spin state (*S* = 1/2) of the Fe^III^S_3_O_3_ site and the high spin state (*S* = 2) of the Fe^II^O_6_ site, which reflects on the presence of two quadrupole doublets assigned to the Fe^II^ and Fe^III^ sites in both of the ^57^Fe Mössbauer spectra of (*n*-C_4_H_9_)_4_N[^57^Fe^II^Fe^III^(mto)_3_] and (*n*-C_4_H_9_)_4_N[Fe^II^^57^Fe^III^(mto)_3_].

## 5. Experimental

### 5.1. Synthesis

#### 5.1.1. Cations

(*n*-C_n_H_2n+1_)_4_NBr (n = 3-6) were commercially available. (*n*-C_m_H_2m+1_)_3_(*n*-C_n_H_2n+1_)NI ((m, n) = (2, 6), (3, 5), (5, 3) and (6, 2)) were prepared according to reference [74]. A solution of tri-*n*-propyl amine and equivalent molar amount of 1-penthyl iodide in 2-butanon was refluxed for 12 hours, then evaporated and recrystallized from methanol to give white microcrystalline solid of (*n*-C_3_H_7_)_3_(*n*-C_5_H_11_)NI. The other compounds were prepared in the similar way with employment of appropriate alkyl amine and alkyl iodide.

Cationic spyropiran was obtained according to reference [42]. Spiro[2H-indole-2,2’-[2H]pyrano[3,2-b]pyridinium],1,3-dihydro-1,3,3,5’,6’-tetramethyl (SP-Me). 3-hydroxy-6-methyl-2-pyridinecarboxaldehyde and equivalent molar amount of 2-methylene-1,3,3-trimethylindoline were refluxed for 3 h in ethanol. The ethanol was evaporated and then water was added to the reaction mixture. The mixture was stirred for several hours and the palely colored precipitate was filtered, washed with water and dried in vacuo. Following this, Spiro[2H-indole-2,2’-[2H]pyrano[3,2-b]pyridinium],1,3-dihydro-1,3,3,5’,6’-pentamethyl iodide ((SP-Me)I) was prepared. SP-Me and 10-times molar amount of methyl iodide were refluxed overnight in THF. The solution was allowed to cool down to room temperature and the yellow microcrystalline solid was filtered, washed with THF, and dried in vacuo.

#### 5.1.2. Ligands and iron mononuclear salt

KBa[Fe^III^(dto)_3_]·3H_2_O was prepared in a way similar to that reported in the literature [4,75]. The di-n-decyl dithiooxalate, (n-C_10_H_21_)_2_(C_2_O_2_S_2_), was obtained by the reaction of oxalyl dichloride (COCl)_2_ and 1-decane thiol of 1 : 2. Potassium dithiooxalate, K_2_C_2_O_2_S_2_, was prepared by the reaction of potassium hydrogensulfide, KHS, and (n-C_10_H_21_)_2_(C_2_O_2_S_2_) of 2:1 in methanol. After filtration, the K_2_C_2_O_2_S_2_ was washed well with methanol to remove the KHS, then it was dried in vacuo. Following this step, Fe(NO_3_)_3_·10H_2_O was treated with three equivalent molar of K_2_C_2_O_2_S_2_ in cold water and the solution was filtered to remove iron sulfide. Then excessive amount of BaBr_2_·2H_2_O (2.50 g, 0.17 mol) was added to the dark purple solution, and KBa[Fe^III^(dto)_3_]·3H_2_O precipitated. The salt was recrystallized from water and dried for one day.

Potassium trithiooxalate was synthesized according to the previous report [76]. After stirring 2 hours for trichloroacetic acid phenyl ester with KHS in methanol with 50 °C, potassium trithiooxalate, K_2_(tto), was given as a pale yellow powder. K_3_[Fe^III^(tto)_3_] salt was obtained from a mixture of potassium trithiooxalate and Fe(NO_3_)_3_ in water.

To obtain the K_2_(mto), equivalent amount of oxalyl-diethyl and KHS were dissolved in ethanol, then the solution was refluxed for 24 hours. The solvent was removed by evaporation. The yellow precipitate was washed with ether and dissolved in fresh ethanol. To this, a solution of potassium hydroxide in ethanol was added dropwise and the solution was allowed to stir for an additional 15 minutes while a milky suspension developed. After cooling the solution at 0 °C, K_2_(mto) was isolated by suction filtration and washed several times with ether. Following this, a methanol solution of iron nitrate was added to a methanol solution of K_2_(mto). After stirring for 5 minutes, [Fe^III^(mto)_3_] solution was given. This solution was filtered once to remove solid impurities. The solution was used for the preparation of the complexes without further purification.

#### 5.1.3. Iron mixed valence complexes

Single crystals of (n-C_3_H_7_)_4_N[Fe^II^Fe^III^(dto)_3_] were grown by a diffusion method. KBa[Fe^III^(dto)_3_]·3H_2_O, FeCl_2_·4H_2_O and ascorbic acid (0.2 g) were placed at one bottom of H-tube while (n-C_3_H_7_)_4_NBr was kept at the other bottom. Then the reaction cell was filled with fresh methanol–water mixture of 10:3 ratio. Single crystals of (n-C_3_H_7_)_4_N[Fe^II^Fe^III^(dto)_3_] were obtained as black needles after a few days.

A powder sample was prepared as follows. A solution of KBa[Fe(dto)_3_]·6H_2_O in a methanol-water mixture was stirred. To this, a solution of FeCI_2_·4H_2_O and (n-C_3_H_7_)_4_NBr in a methanol-water mixture was added. In this way, powder sample of (n-C_3_H_7_)_4_N[Fe^II^Fe^III^(dto)_3_] was obtained as black colored precipitate.

Other iron mixed valence complexes were synthesized in a way similar to the preparation of powder (n-C_3_H_7_)_4_N[Fe^II^Fe^III^(dto)_3_] by using appropriate cation and bridging ligand.

### 5.2. Crystal Structure

The powder X-ray diffraction (PXRD) profiles were measured with Rigaku, Multiflex using Cu *K_□_* radiation at room temperature. The PXRD for a part of dto complexes were taken at the BL02B2 beam line of SPring-8 with the wavelength of 1.001 Å. For the single crystal of the (n-C_3_H_7_)_4_N[Fe^II^Fe^III^(dto)_3_], the X-ray crystal structure analysis was carried out by using of a Rigaku, RAXIS-RAPID Imaging Plate diffractometer equipped with graphite-monochromated Cu *K_□_* radiation.

### 5.3. Measurements of Physical Properties

The static magnetic susceptibility was measured by a Quantum Design, MPMS-5 SQUID susceptometer under 5000 Oe T. The magnetic susceptibility obtained was corrected for the background and the core diamagnetism. The diamagnetic correction constituting atoms was carried out by using Pascal's constants. The zero field cooled magnetization (ZFCM) and field cooled magnetization (FCM) were also measured for the investigation of the ferromagnetic phase below the ordering temperature under 30 Oe. The remnant magnetization (RM) was measured in the same temperature region under zero field.

For the ^57^Fe Mössbauer spectroscopic measurement, ^57^Co in Rh was used as a Mössbauer source. The spectra were calibrated by using the six lines of a body-centered cubic iron foil (□-Fe), the center of which was taken as zero isomer shift. The hyperfine parameters were obtained by least-squares fitting to Lorentzian line shapes. The ^57^Fe Mössbauer spectroscopy was also performed in the low temperature region with an Iwatani Industrial Gases Corporation, cryogenic refrigerator set, Cryomini and MiniStat.

The UV-vis spectra were monitored with a JASCO MSV-370 by KBr method at room temperature and 70 K. Temperature was controlled by an Oxford CRYOMINI and an Oxford ITC503S temperature controller. An Asahi Spectra Co. LAX-101 (350 nm, 40 mW/cm^2^) Xe lamp as the UV source and a Hoya-Schott, MEGALIGHT 100-S (white light, 600 mW/cm^2^) as the visible light source were used, when the light irradiation to the photoisomerization in the complexes was needed.

A μSR experiment was performed at the RIKEN-RAL Muon Facility in the United Kingdom by using a pulsed positive surface-muon beam. The time dependence of the asymmetry parameter of muon spin polarization (μSR time spectrum) was measured in ZF and a LF. The initial muon spin polarization is in parallel with the beam line and the direction of the LF is parallel to the internal muon spin polarization. The depolarization processes were analyzed by Kubo-Toyabe function and so forth.

## 6. Conclusion

Assembled hetero-molecular systems such as organic-inorganic hybrid system have the possibility to undergo significant concert phenomena as a whole system through the hetero-molecular interaction between the constituent elements. From this viewpoint, we have developed organic-inorganic hybrid systems, A[Fe^II^Fe^III^X_3_] (A = (*n*-C_n_H_2n+1_)_4_N, spiropyran; X = dto(C_2_O_2_S_2_), tto(C_2_OS_3_), mto(C_2_O_3_S)), and have investigated their multifunctional properties coupled with spin, charge and photon.

In (*n*-C_n_H_2n+1_)_4_N[Fe^II^Fe^III^(dto)_3_](n = 3,4), a new type of phase transition called charge transfer phase transition (CTPT) takes place around 120 K, where the thermally induced charge transfer phase transition reversibly occurs around 120 K in order to minimize the free energy in the whole system. In the high temperature phase (HTP), the Fe^III^ (*S* = 1/2) and Fe^II^ (*S* = 2) sites are coordinated by six S atoms and six O atoms, respectively. In the low temperature phase (LTP), on the other hand, the Fe^III^ (*S* = 5/2) and Fe^II^ (*S* = 0) sites are coordinated by six O atoms and six S atoms, respectively. The driving force responsible for the CTPT is considered to be the difference in spin entropy between HTP and LTP. At the CTPT, the iron valence state is dynamically fluctuated with a frequency of about 0.1 MHz, which was confirmed by means of muon spin relaxation. Moreover, (*n*-C_3_H_7_)_4_N[Fe^II^Fe^III^(dto)_3_] behaves as a ferromagnet with *T*_C_ = 7 K. The charge transfer phase transition and the ferromagnetic transition remarkably depend on the hexagonal ring size in the 2D honeycomb network structure of [Fe^II^Fe^III^(dto)_3_]_∞_. The increase of cation size expands the honeycomb ring, which stabilizes the higher temperature phase with Fe^II^ (*S* = 2) and Fe^III^ (*S* = 1/2). In order to control the magnetic properties and the electronic state in the dto-bridged iron mixed-valence system by means of photo-irradiation, we have synthesized a photo-sensitive organic-inorganic hybrid system, (SP)[Fe^II^Fe^III^(dto)_3_] (SP = spiropyran), and have found that the UV irradiation of 350 nm changes the structure of spiropyran from the closed form to the open one in (SP)[Fe^II^Fe^III^(dto)_3_], which induces the charge transfer phase transition from the lower temperature phase to the higher temperature phase in the two-dimensional honeycomb network structure of [Fe^II^Fe^III^(dto)_3_]_∞_, and enhances the ferromagnetic transition temperature.

In the case of (*n*-C_3_H_7_)_4_N[Fe^II^Fe^III^(tto)_3_] (tto = C_2_OS_3_), a ferromagnetic transition takes place at 9.5 K. From the analysis of ^57^Fe Mössbauer spectra, it is concluded that the LTP with Fe^II^(*S* = 0) and Fe^III^(*S* = 5/2) is stable in the whole measuring temperature between 300 K and 5 K.

In the case of (*n*-C_n_H_2n+1_)_4_N[Fe^II^Fe^III^(mto)_3_](mto = C_2_O_3_S), a ferrimagnetic transition takes place at 38 K. In this system, a rapid spin equilibrium between the high-spin state (*S* = 5/2) and the low-spin state (*S* = 1/2) at the Fe^III^O_3_S_3_ site takes place in a wide temperature range, which induces the valence fluctuation of the FeS_3_O_3_ and FeO_6_ sites in (*n*-C_4_H_9_)_4_N[Fe^II^Fe^III^(mto)_3_] through the ferromagnetic coupling between the low spin state (*S* = 1/2) of the Fe^III^S_3_O_3_ site and the high spin state (*S* = 2) of the Fe^II^O_6_ site, which reflects on the presence of two quadrupole doublets assigned to the Fe^II^ and Fe^III^ sites in both of the ^57^Fe Mössbauer spectra of (*n*-C_n_H_2n+1_)_4_N[^57^Fe^II^Fe^III^(mto)_3_] and (*n*-C_n_H_2n+1_)_4_N[Fe^II^^57^Fe^III^(mto)_3_].
